# DNTTIP1 drives leukaemogenesis through MiDAC‐mediated epigenetic silencing of BMF

**DOI:** 10.1002/ctm2.70603

**Published:** 2026-01-28

**Authors:** Ruolin Xiu, Yuzhu Ma, Yueying Gao, Yao Chen, Xinyu Li, Yue Wu, Meiling Sun, Qizhao Li, Yanhong Zhao, Shuqian Xu, Shengjin Fan, Yongsheng Li, Huitao Fan

**Affiliations:** ^1^ Department of Critical Care Medicine Department of Hematology NHC Key Laboratory of Cell Transplantation Key Laboratory of Hepatosplenic Surgery of Ministry of Education, The First Affiliated Hospital of Harbin Medical University Harbin China; ^2^ Department of Hematology The First Affiliated Hospital of Harbin Medical University Harbin China; ^3^ State Key Laboratory of Frigid Zone Cardiovascular Diseases School of Interdisciplinary Medicine and Engineering Harbin Medical University Harbin China; ^4^ Department of Hematology Shandong Key Laboratory of Hematological Diseases and Immune Microenvironment, Shandong Provincial Clinical Research Center for Hematological Diseases Qilu Hospital of Shandong University Jinan China; ^5^ State Key Laboratory of Frigid Zone Cardiovascular Diseases Harbin Medical University Harbin China

**Keywords:** acute leukaemia, apoptosis, autophagy, BMF, DNTTIP1, MiDAC

## Abstract

**Background:**

Acute leukaemia is a highly aggressive malignancy with significant unmet therapeutic needs, partly due to epigenetic dysregulation. Here, we uncover deoxynucleotidyl transferase terminal‐interacting protein 1 (DNTTIP1) within the mitotic deacetylase complex (MiDAC) as a previously unrecognised epigenetic regulator crucial for leukaemic cell survival and elucidate its mechanistic and translational significance.

**Methods:**

Using cellular, biochemical, and genetic perturbations, coupled with validation in multiple in vivo leukaemia mouse models, we characterised DNTTIP1's role in acute leukaemia. An integrated multi‐omics analysis incorporating RNA‐seq, cleavage under targets and tagmentation (CUT&Tag) and assay for transposase‐accessible chromatin using sequencing (ATAC‐seq) revealed that DNTTIP1 recruits histone deacetylase 1/2 (HDAC1/2) to silence BCL2‐modifying factor (BMF) and drive leukaemogenesis, validated by chromatin immunoprecipitation quantitative PCR (ChIP‐qPCR). Drug synergy assays identify poly(ADP‐ribose) polymerase (PARP)/HDAC/BCL2 inhibitor combinatorial efficacy.

**Results:**

DNTTIP1 depletion impaired MiDAC recruitment in acute leukaemia, leading to histone H3 lysine 27 (H3K27) hyperacetylation at the BMF promoter and reactivating this effector. Upregulated BMF disrupted BCL2‐mediated survival, triggering coordinated autophagy and apoptosis. Combined HDAC1/2 and BCL2 inhibition exerts synergistic anti‐leukaemic effects, a therapeutic strategy currently under clinical evaluation. Further, PARP inhibition profoundly enhanced this synergy by impairing DNA damage repair, unveiling a novel triple‐combination strategy.

**Conclusions:**

Our work defines the DNTTIP1‒HDAC1/2‒BMF axis as a pivotal epigenetic vulnerability in acute leukaemia and provides preclinical rationale for targeting this axis. These findings offer a validated biological framework for advancing this targeted combination therapy into clinical trials.

**Key points:**

- DNTTIP1 is overexpressed in acute leukaemia and associated with poor prognosis.
- DNTTIP1 acts as a scaffold for the MiDAC complex, recruiting HDAC1/2 to silence BMF and inhibit leukaemic cell death.
- Pharmacological disruption of the DNTTIP1–HDAC1/2–BMF axis impairs leukaemogenesis.

## Graphical abstract

DNTTIP1 cooperates with SP1 to recruit HDAC1/2, leading to BMF silencing and reduced competitive binding of BMF to BCL2.This suppresses autophagy and apoptosis, promoting leukaemic cell survival. Targeting the DNTTIP1‐HDAC1/2‐BMF axis reactivates BMF and induces anti‐leukaemic effects. The “HDACi+BCL2i+PARPi” triple combination shows potent efficacy and safety in preclinical models.

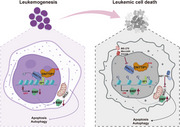

## 1 INTRODUCTION

Acute leukaemias are aggressive haematologic neoplasms characterised by uncontrolled proliferation of immature haematopoietic precursors that disrupt normal bone marrow (BM) function.^1^ Accumulating evidence implicates the critical role of epigenetic dysregulation, particularly aberrant histone modifications, in malignant transformation and disease progression.^2^, ^3^, ^4^ These malignancies exhibit distinct DNA methylation abnormalities and dysregulated histone deacetylase (HDAC) activity,^5^ highlighting epigenetic modifiers as promising therapeutic targets. Studies have demonstrated that HDACs orchestrate the expression of genes governing cell cycle progression, differentiation, and apoptosis, processes frequently dysregulated in leukaemogenesis.^6^ While DNA hypomethylating agents have exhibited remarkable therapeutic efficacy,^7^, ^8^ the therapeutic potential of HDAC inhibitors (HDACi) in acute leukaemia remains incompletely defined and context dependent. However, recent studies have reported that novel HDACi can induce apoptosis and constrain autophagy in leukaemic cells,^9^ and HDACi have shown promise in treating preleukaemic conditions and leukaemia driven by JAK2^V617F^ mutations.^10^ Multiple ongoing clinical trials are currently evaluating the therapeutic impact of HDACi targeting HDAC dysregulation across various haematologic malignancies, including acute myeloid leukaemia (AML), acute lymphoblastic leukaemia (ALL), multiple myeloma and lymphoma.^11^, ^12^, ^13^, ^14^, ^15^ Further insights into HDACs will uncover the molecular drivers of acute leukaemia, enabling more rational combination therapy approaches.

HDACs exhibit minimal catalytic activity as isolated enzymes but undergo conformational changes upon incorporation into multi‐protein complexes, enhancing deacetylase efficiency and enabling locus‐specific recruitment through complex‐specific targeting domains.^16^, ^17^ Deoxynucleotidyl transferase terminal‐interacting protein 1 (DNTTIP1) acts as a critical scaffold protein indispensable for the assembly of the mitotic deacetylase complex (MiDAC), one of the seven known HDAC core complexes,^18^ mediating the formation of a catalytically active tetramer with distinct functional properties. MiDAC demonstrates the highest nucleosome deacetylation efficiency among all HDAC complexes,^19^ directly implicating DNTTIP1 as a master regulator of chromatin accessibility and epigenetic control. In addition to its role in MiDAC assembly, DNTTIP1 functions as a DNA‐binding protein that interacts with terminal deoxynucleotidyltransferase (TdT). This interaction enhances TdT activity and promotes template‐independent DNA polymerisation, a process critical for V(D)J recombination and antigen receptor diversity.^20^ Beyond these well‐established roles, growing evidence indicates that DNTTIP1 also participates in cell cycle progression and DNA repair,^21^, ^22^ yet its precise mechanistic contributions remain poorly understood. Although DNTTIP1 dysregulation has been implicated in various cancers,^23^, ^24^ its specific role in leukaemia and potential contribution to leukaemogenesis remain entirely unexplored.

BCL2‐modifying factor (BMF) encodes a BH3 (BCL2 homology 3)‐only protein, a pro‐apoptotic member of the BCL2 protein family that binds and neutralises pro‐survival BCL2 proteins.^25^, ^26^ As the first identified gene promoting prolonged cell survival, BCL2 underscores resistance to cell death as a key tumour hallmark driving tumour progression and therapeutic resistance. Through protein‒protein interactions, BCL2 concurrently neutralises the pro‐apoptotic effectors BAX/BAK and blocks the autophagy regulator Beclin‐1, thereby coordinating cell survival pathways.^27^, ^28^ This dual role positions BCL2 as both an anti‐apoptotic protein and an anti‐autophagic protein. However, the presence of sufficient BH3‐only proteins can saturate BCL2, leading to the release of BAX/BAK and Beclin‐1 from sequestration and promoting apoptosis and autophagy.^29^ Given the frequent dysregulation of these pathways in leukaemia,^30^ they have become important therapeutic targets for haematologic malignancies.^31^, ^32^

Here, we report that DNTTIP1, a key scaffold subunit of MiDAC, drives oncogenic activity in acute leukaemia by recruiting HDAC1/2 to maintain histone H3 lysine 27 (H3K27) deacetylation, leading to BMF silencing. Genetic or pharmacological disruption of the DNTTIP1‒HDAC1/2‒BMF axis triggers a coordinated cell death response in acute leukaemia, engaging both apoptotic and autophagic pathways. These findings identify a previously unrecognised vulnerability for therapeutic intervention.

## 2 MATERIALS AND METHODS

### 2.1 Cell lines and cell culture

The human leukaemia cell lines (RS4;11, MV4‐11, THP‐1, MOLM‐13 and K562) were purchased from SAIBAIKANG and cultured in RPMI‐1640 medium (VivaCell) supplemented with 10% foetal bovine serum (FBS; Vazyme), 1% penicillin/streptomycin (NCM Biotech). HEK293T cell line was purchased from the ATCC and maintained in Dulbecco's modified Eagle medium (VivaCell) supplemented with 10% FBS. Cell cultures were maintained at 37°C in a 5% CO_2_ humidified atmosphere. Cell line identity was confirmed via short tandem repeat analysis, and mycoplasma contamination was periodically tested, ensuring experimental reliability.

### 2.2 Patient samples

BM samples were collected from patients with an initial diagnosis of acute leukaemia (AML and ALL) and healthy donors at the First Affiliated Hospital of Harbin Medical University. This study was conducted with approval by the Research Ethics Committee and the Ethics Committee of the First Affiliated Hospital of Harbin Medical University (2022189) and adhered to the principles of the Declaration of Helsinki. Mononuclear cells were isolated from freshly collected BM samples (acute leukaemia patients and healthy donors) using Ficoll gradient centrifugation (TBD Sciences) following the manufacturer's protocol. Patient characteristics are summarised in Table S1.

### 2.3 Reagents and antibodies

Drugs for in vitro studies were stored at ‒20°C at 10 mM in Dimethyl sulfoxide (DMSO) and listed as follows: cytarabine (Ara‐C) (TargetMol, T1272), daunorubicin hydrochloride (DNR) (TargetMol, T1511), doxorubicin (DOX) (TargetMol, T1456), vincristine sulphate (VCR) (TargetMol, T1270), venetoclax (ABT‐199) (Selleckchem, S8048), entinostat (MS‐275) (Selleckchem, S1053), tacedinaline (CI‐994) (Selleckchem, S2818), 5‐azacytidine (5‐AzaC) (MCE, HY‐10586), Olaparib (TargetMol, T3015), Merck60 (BRD‐6929) (MCE, HY‐100719), RGFP966 (MCE, HY‐13909) and FK228 (Romidepsin) (MCE, HY‐15149). Antibodies are summarised in Table S2.

### 2.4 Plasmid, lentivirus production and transduction

The DNTTIP1 overexpression (OE) lentiviral vector was constructed by cloning the CDS region of DNTTIP1 with a 3×HA tag into the pLVX‐IRES‐neo vector. For truncation vectors, a series of deletion of the DNTTIP1 amino acid (aa) sequence were designed based on its functional domain annotation: full‐length (aa 1‒317), N‐terminal domain (aa 1‒56), dimerisation domain (DD, aa 57‒148), a middle region (aa 149‒197) and the C‐terminal domain encoding the Ski/Sno/Dac motif (aa 198‒317). Gene‐specific primers for amplifying the full‐length CDS and truncated fragments were designed. Truncated fragments corresponding to different domain deletions and the full‐length DNTTIP1 CDS were amplified via PCR using the synthesised cDNA as the template. The BMF OE lentiviral vector was constructed by cloning the full‐length CDS of human BMF with a Flag tag into the pLenti‐GIII‐CMV vector, purchased from Applied Biological Materials (ABM). Short hairpin RNAs (shRNA) targeting either DNTTIP1 (shDNTTIP1) or HDAC1 (shHDAC1) were designed and cloned into lentiviral vector pLKO.1‐puro (addgene plasmid 10878) according to the manufacturer's instructions. Two independent small interfering RNAs (siRNAs) targeting DNTTIP1 (siDNT_1 and siDNT_2) and a non‐targeting control siRNA (siControl) were purchased from Hippobio. The shRNA plasmids pLenti‐U6‐shBMF‐CMV‐GFP‐2A‐Hygro and pLenti‐U6‐shHDAC2‐CMV‐Hygro (targeting BMF and HDAC2, respectively) were purchased from ABM. The CRISPR/Cas9 expression vectors pLenti‐U6‐sgSP1‐SFFV‐Cas9‐2A‐Hygro (targeting SP1) and pLenti‐U6‐sgDNTTIP1‐SFFV‐Cas9‐2A‐Puro (targeting DNTTIP1) were also obtained from ABM. The oligo sequences used to construct the plasmid are listed in Table S3.

Lentivirus was produced by transfecting HEK293T cells with target gene plasmids and packaging plasmids psPAX2 (Addgene, 12260) and pMD2.G (Addgene, 12259) using PEI MAX (PolyScience, 24765‐1) in OPTI‐MEM medium (Gibco, 31985‐070). Viral supernatants were collected at 48 and 72 h post‐transfection, filtered through a 0.45 µM strainer, and then mixed with 10 µg/mL polybrene (Biosharp, BL628A) for centrifugation at 2500 rpm for 90 min. Medium were replaced with fresh medium 24 h post‐infection. Stable DNTTIP1 knockdown (KD)/knockout (KO), HDAC1‐KD and BMF OE cell lines were selected and maintained in medium containing 2 µg/mL puromycin (RHAWN, R032317). Meanwhile, DNTTIP1 OE cells were selected with 400‒800 µg/mL G‐418 (MCE, HY‐17561), whereas BMF‐KD, HDAC2‐KD and SP1‐KO stable cells were selected with 300‒600 µg/mL hygromycin B (Solarbio, R032317). Transfection of siRNA was performed using Lipofectamine RNAiMAX (Invitrogen, 13778075) according to the manufacturer's instructions.

### 2.5 Pull‐down assay

Glutathione S‐transferase (GST)/His pull‐down assays were performed using a pull‐down kit (Biolinkedin, IK‐2004) following the manufacturer's instructions. Briefly, 5 µg GST‐tagged HDAC1 (SignalChem, H83‐30G) and 5 µg His‐tagged DNTTIP1 (Solarbio, P05485) were mixed with 20 µL pre‐washed beads (GST‐labelled magnetic beads for GST pull‐down; NTA‐Ni beads for His pull‐down) in pull‐down buffer. Controls included GST protein (MCE, HY‐P70270) substituting GST‐HDAC1 (GST pull‐down) and empty NTA‐Ni beads (His pull‐down). All mixtures were rotated overnight at 4°C, beads collected via magnetic rack, washed three times with wash buffer, and bound proteins eluted with SDS sample buffer for Western blot analysis.

### 2.6 Chromatin immunoprecipitation quantitative PCR

Chromatin immunoprecipitation (ChIP) assays were performed using a ChIP assay kit (Beyotime, P2083S) according to the manufacturer's instructions. Briefly, cells were cross‐linked with 1% formaldehyde (Sigma) at 37°C for 10 min, followed by quenching with glycine. Cells were then resuspended in lysis buffer after being washed with phosphate buffered saline (PBS) supplemented with a protease inhibitor cocktail. Chromatin‒antibody complexes were efficiently precipitated using Protein A/G Magnetic Beads. Following elution, the chromatin was subjected to reverse crosslinking at 65°C for a duration of 4 h. The purified DNA was then processed using QIAquick PCR Purification kit (Qiagen, 28140) and amplified via qPCR with locus‐specific primer sets detailed in Table S3. Chromatin immunoprecipitation quantitative PCR (ChIP‐qPCR) calculations were conducted as previously described.^33^

### 2.7 Proximity ligation assay

Proximity ligation assay (PLA) was performed following the NaveniFlex Cell MR Red kit (Navinci, NC.MR.100 Red) protocol. Briefly, cells were seeded onto chamber slides, fixed with 4% paraformaldehyde (30 min, RT), permeabilised with .1% Triton X‐100 in PBS and blocked (60 min, 4°C) before sequential incubations with primary antibodies (overnight, 4°C), Navenibody (60 min, 37°C) and ligase solution (120 min, 37°C). After washing, samples were mounted with Hoechst‐containing antifade medium and imaged on a confocal microscope (Carl Zeiss, LSM 900).

### 2.8 Comet assay

DNA damage was quantified using an alkaline comet assay (Beyotime, C2041M) adapted for haematopoietic cells. Briefly, 1 × 10^5^ cells were embedded in .7% low‐melting‐point agarose on pre‐coated comet slides using a double‐layer method. The slides were then lysed in ice‐cold alkaline buffer (pH 10.0, 10% DMSO) for 2 h at 4°C, followed by DNA denaturation in electrophoresis buffer (200 mM NaOH and 1 mM EDTA) for 1 h. Electrophoresis was performed at 1 V/cm for 30 min (4°C, protected from light). After neutralisation (.4 M Tris‒HCl, pH 7.5) and propidium iodide staining, a minimum of 100 comets per sample were imaged using fluorescence microscopy. DNA damage was analysed using ImageJ, with tail DNA (%) and tail moment (Olive tail moment, calculated as tail length × fraction of DNA in the tail) as the primary metrics.

### 2.9 Cell line‐derived xenograft model

Female NCG mice (6 weeks old) from GemPharmatech were acclimated for 7 days under pathogen‐free conditions before intravenous injection of 5 × 10^6^ luciferase‐labelled RS4;11 control cells (pLKO) or DNTTIP1‐KD cells (shDNT_4) via the tail vein. Mice received an intraperitoneal injection of D‐Luciferin (Gold Biotechnology, 18567‐5MG) 2 weeks post‐transplantation and imaged weekly at 15 min post‐injection under anesthesia using IndiGo (Berthold).

### 2.10 Patient‐derived xenograft models

Primary ALL cells were isolated from patient BM (Table S1) and intravenously transplanted into sublethally irradiated (2.5 Gy) NCG mice aged 6 weeks. Splenic ALL cells harvested from primary recipients were serially transplanted into subsequent irradiated recipients. For in vitro experiments, patient‐derived xenograft (PDX) cells were maintained in vitro and transfected with RNAi constructs to generate stable DNTTIP1‐KD cells. For in vivo experiments, PDX cells were intravenously injected into sublethally irradiated (2.5 Gy) NCG mice. Two weeks following cell inoculation, after confirmation of successful engraftment defined as human CD45‐positive (hCD45+) cells exceeding 1% in the peripheral blood (PB) via flow cytometry, mice were randomly allocated into five groups (*n* = 8 per group) and treated with vehicle, FK228 (1.5 mg/kg, i.p., twice weekly), ABT‐199 (100 mg/kg, p.o., daily), FK228 combined with ABT‐199, or FK228 plus ABT‐199 and Olaparib (50 mg/kg, i.p., daily) for three consecutive weeks. Six weeks after cell inoculation, three mice per group were sacrificed for phenotypic analysis, while the remaining five mice in each group were monitored for survival.

### 2.11 The Cancer Genome Atlas and Therapeutically Applicable Research to Generate Effective Treatments dataset analysis

Publicly available human transcriptome data for leukaemia were obtained from The Cancer Genome Atlas (TCGA) and Therapeutically Applicable Research to Generate Effective Treatments (TARGET). Transcript expression levels were normalised using the transcripts per million method and subsequently analysed. Differential expression between groups was assessed using the Wilcoxon rank‐sum test. To quantify the HDAC transcriptional signature, single‐sample gene set enrichment analysis (ssGSEA) was performed to calculate an HDAC enrichment score based on the mRNA expression profiles of the 18 members of the HDAC family.^34^ This score reflects the collective enrichment of HDAC transcripts within individual samples. Survival outcomes were compared between groups using Kaplan‒Meier survival curves with log‐rank tests, where a *p *< .05 was considered statistically significant. Correlations between HDACs genes expression and other genes were evaluated using Pearson's method.

### 2.12 Statistical analysis

For most experiments, the sample size was set at *n* ≥ 3 biological replicates per group. In animal studies, group sizes were determined based on preliminary data to ensure adequate statistical power for detecting biologically relevant effects. Transcriptomic data were processed using R software (version 4.4.1) with two‐tailed Wilcoxon rank‐sum test for non‐normally distributed group comparisons, and survival analyses were performed using Kaplan‒Meier curves with log‐rank test via R package survival (v.3.7.0) and survminer (v.0.5.0). Other statistical analyses were conducted in GraphPad Prism software, including unpaired two‐tailed Student's *t*‐test for comparison between two groups, one‐way ANOVA with Dunnett's for multiple comparisons tests. The Pearson tests were performed for correlation analysis. Data are presented as mean ± standard deviation, and statistical significance was defined as *p *< .05 for all analyses (^*^ *p *< .05, ^**^ *p *< .01, ^***^ *p *< .001, ^****^ *p *< .0001).

## 3 RESULTS

### 3.1 DNTTIP1 is a key interactor of HDAC1/2 and serves as a prognostic marker in acute leukaemia

**FIGURE 1:**
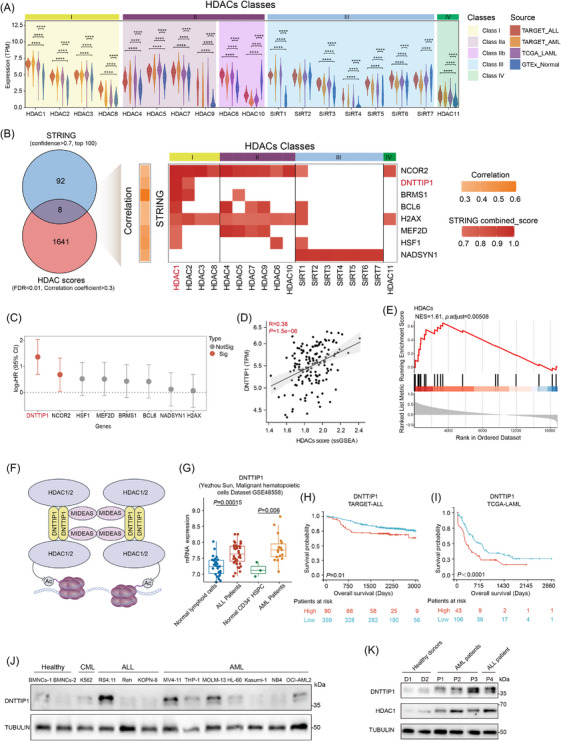
Deoxynucleotidyl transferase terminal‐interacting protein 1 (DNTTIP1) is a key interactor of histone deacetylase 1 (HDAC1) and serves as a prognostic marker in acute leukaemia. (A) Expression levels of HDACs classes from The Cancer Genome Atlas (TCGA) and Therapeutically Applicable Research to Generate Effective Treatments (TARGET) datasets. Transcript per million (TPM) was used to quantify gene expression. (B) Venn diagram (left) showing the overlap of the top 100 genes interacting with HDACs identified from the STRING dataset (confidence > .7) and the HDAC scores derived from single‐sample gene set enrichment analysis (ssGSEA) of the TCGA dataset (False discovery rate [FDR] < .01, correlation coefficient > .3). Heatmap (right) illustrates the correlation between eight genes and the HDACs score (indicated in orange), as well as the interaction network confidence with HDACs (indicated in red). (C) Cox proportional hazards regression analysis reveals the prognostic value of the genes. Dots represent log_2_‐transformed hazard ratio (HR) and horizontal lines indicate 95% confidence intervals (95% CIs). Genes are ranked according to the magnitude of their HR. (D) Pearson correlation analysis demonstrating correlations between DNTTIP1 expression and HDACs score (*R* = .38, *p *= 1.5e−06). (E) GSEA showing DNTTIP1 enrichment in HDAC‐related pathways (Normalized enrichment score [NES] = 1.61, *p*.adjust = .00508). (F) Schematic representation of the mitotic deacetylase complex (MiDAC). (G) Expression analysis of DNTTIP1 in dataset GSE48558. (H and I) Survival analysis of DNTTIP1 in TARGET (K) and TCGA (L) datasets. Survival differences were evaluated using the log‐rank test, with a significance threshold of *p *< .05. (J) Western blotting (WB) analysis of DNTTIP1 protein expression in acute leukaemia cell lines, and healthy donor‐derived bone marrow mononuclear cells (BMNCs) as a normal control. A representative blot is shown. (K) Western blot analysis of DNTTIP1 and HDAC1 in BMNCs from acute leukaemia patients (*n* = 4) and healthy donors (*n* = 2). A representative blot is shown.

To examine altered HDACs in acute leukaemia, we performed a comprehensive genomic profiling of all 18 human deacetylases, phylogenetically classified into the HDAC and sirtuin superfamilies.^5^ Through integrated analysis of three major clinical genomics repositories, TCGA dataset, TARGET dataset and NCBI Gene Expression Omnibus dataset, we identified HDAC1 as one of the most highly expressed deacetylases, with statistically significant upregulation in acute leukaemia samples compared to non‐malignant haematopoietic cells (Figures 1A and S1A). Further analysis of the CRISPR/Cas9 screening dataset of over 1000 cancer cell lines revealed that HDAC1 is more critical for the survival of lymphoid leukaemia/lymphoma cells than for other cancer cell types (Figure S1B). A combined analysis of STRING protein‒protein interaction networks and TCGA transcriptomic data (analysed via ssGSEA) revealed eight genes associated with HDACs (Figures 1B and S1C,D). Notably, DNTTIP1 exhibited exclusive interaction with HDAC1 and HDAC2, distinguishing it among these candidates. Cox proportional hazards regression analysis of these eight candidate genes demonstrated that elevated expression levels of DNTTIP1 and NCOR2 were significantly associated with poor survival outcomes in acute leukaemia (Figure 1C). Intriguingly, DNTTIP1 exhibited the strongest association with adverse prognosis, solidifying its role as both an independent prognostic biomarker and a selective HDAC1/2 co‐regulator in haematologic malignancies. As anticipated, DNTTIP1 expression positively correlated with HDACs scores (Figure 1D). Supporting this observation, GSEA revealed significant involvement of DNTTIP1 in HDAC‐related pathways (Figure 1E).

We therefore targeted DNTTIP1, a core scaffold protein essential for MiDAC assembly and stability. Unlike other HDAC complexes, MiDAC exhibits a unique structural organisation (Figure 1F), in which DNTTIP1 and mitotic deacetylase associated SANT domain protein (MIDEAS) form a central platform that recruits four HDAC1/2 molecules—the highest stoichiometry reported for all HDAC complexes.^21^ Analysis of public leukaemia datasets revealed significant upregulation of DNTTIP1 in acute leukaemia patients compared to healthy controls (Figure 1G). Notably, elevated DNTTIP1 expression showed the strongest association with reduced overall survival (Figure 1H,I), whereas other candidate genes, except NCOR2, lacked prognostic significance (Figure S1E‒K), corroborating our initial Cox regression analysis. We further validated the upregulation of DNTTIP1 in leukaemic lineages through the Human Protein Atlas database (Figure S1L). At the translational level, Western blot analysis recapitulated robust DNTTIP1 protein expression across a panel of ALL and AML cell lines (Figure 1J). Moreover, primary leukaemia samples exhibited markedly higher DNTTIP1 levels compared to those from healthy donors, with concurrent confirmation of the expression of other MiDAC complex components (HDAC1, HDAC2 and MIDEAS) in these clinical specimens via the same assay (Figures 1K and S1M). Together, these data establish DNTTIP1 as a consistently upregulated biomarker in acute leukaemia and a strong predictor of poor patient outcomes.

### 3.2 DNTTIP1 sustains leukaemogenesis by promoting cell survival and proliferation in acute leukaemia

**FIGURE 2:**
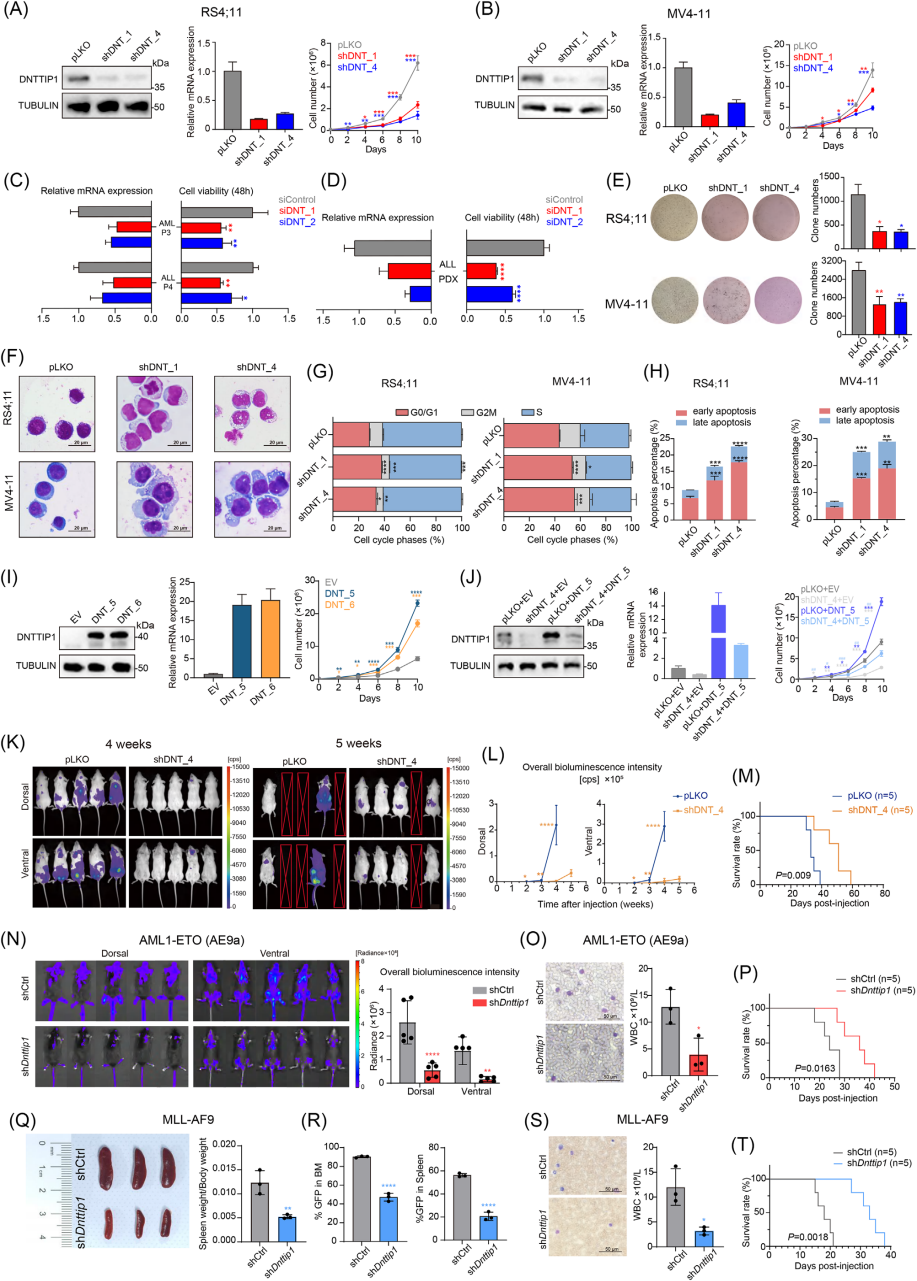
Deoxynucleotidyl transferase terminal‐interacting protein 1 (DNTTIP1) sustains leukaemogenesis by promoting cell survival and proliferation in acute leukaemia. (A and B) Immunoblot (left) and RT‐qPCR (middle) analyses demonstrating DNTTIP1 knockdown using two independent short hairpin RNAs (shRNAs) (shDNT_1 and shDNT_4). Cell counting of RS4;11 (A) and MV4‐11 (B) cells expressing shDNT_1 and shDNT_4 compared with control (pLKO) (right) (*n* = 3). (C and D) Bar chart showing DNTTIP1 knockdown efficiency (left) and proliferation suppression (right) in acute leukaemia bone marrow (BM) cells (C) and B‐cell acute lymphoblastic leukaemia (B‐ALL) patient‐derived xenograft (PDX) cells (D) 48 h post‐siRNA transfection. (E) Colony‐forming unit (CFU) assay of RS4;11 and MV4‐11 cells with DNTTIP1 knockdown (shDNT_1 and shDNT_4) compared with control (pLKO) (*n* = 3) (left). Quantitative analysis of CFU assays (right). (F) Morphological analyses of RS4;11 and MV4‐11 cells following DNTTIP1 knockdown by Cytospin preparation and Wright‒Giemsa staining. Scale bar = 10 µM. (G) Flow cytometric analysis was performed to assess cell cycle distribution following DNTTIP1 knockdown in RS4;11 and MV4‐11 cells (*n* = 3). (H) Flow cytometric analysis of Annexin V/propidium iodide (PI) staining in RS4;11 and MV4;11 cells with DNTTIP1 knockdown (shDNT_1 and shDNT_4) versus control (pLKO) (*n* = 3). (I) Immunoblot (left) and RT‐qPCR (middle) analyses demonstrating DNTTIP1 overexpression using two single clones (DNT_5 and DNT_6). Cell counting of RS4;11 cells overexpressing DNTTIP1 compared with control (EV) (right) (*n* = 3). (J) Immunoblot (left) and RT‐qPCR (middle) analyses demonstrating the rescue effect of DNTTIP1 overexpression (DNT_5) on DNTTIP1 knockdown (shDNT_4). Cell counting of RS4;11 cells with rescued DNTTIP1 expression compared with control (right) (*n* = 3). shDNT_4+DNTTIP1_5 versus pLKO + DNTTIP1_5 is denoted by symbol (#). All groups versus pLKO + EV is denoted by symbol (*). (K) Representative bioluminescence images of NCG mice at the indicated experimental timepoint following xenotransplantation of luciferase‐labelled RS4;11 cells (*n* = 5 per group). ‘X’ denotes deceased subjects (endpoint). (L) The dorsal (left) and ventral (right) imaging bioluminescent signals are depicted. (M) Kaplan‒Meier survival plots were employed to illustrate the survival of mice, with curve comparisons analysed using the log‐rank test (*n* = 5 per group). (N) Representative bioluminescence images of sublethally irradiated C57BL/6J mice at 3 weeks post‐xenotransplantation of luciferase‐tagged AML1‐ETO (AE9a) murine acute leukaemia cells (left). Quantitative analysis of overall bioluminescence intensity, presented as a bar graph (right) (*n* = 5 per group). (O and S) Giemsa staining of peripheral blood (PB) smears from recipient mice transplanted with DNTTIP1‐targeting shRNA (shDNT)‐expressing AML1‐ETO (AE9a) or MLL‐AF9 leukaemia cells (left). White blood cell (WBC) counts of recipient mice at 2 weeks post‐transplantation with AML1‐ETO (AE9a) or MLL‐AF9 leukaemia cells (right) (*n* = 5 per group). (P and T) Kaplan‒Meier survival plots were employed to illustrate the survival of mice, with curve comparisons analysed using the log‐rank test (*n* = 5 per group). (Q) Spleen size (left) and spleen/body weight (right) in mice 2 weeks post‐transplantation with MLL‐AF9 cells stably expressing DNTTIP1‐targeting shRNA or non‐targeting control shRNA (*n* = 3). (R) Percentage of GFP^+^ leukaemic blast cells in BM and spleen of recipient mice at 2 weeks post‐transplantation of MLL‐AF9 cells (*n* = 3). Quantitative data are presented as the mean ± standard deviation (SD) from at least three independent experiments and analysed by Student's *t*‐test. ^*^ *p *< .05, ^**^ *p *< .01, ^***^ *p *< .001, ^****^ *p *< .0001.

To investigate the functional role of DNTTIP1 in acute leukaemia, we employed two independent shRNAs (shDNT_1 and shDNT_4) targeting distinct regions of the DNTTIP1 gene to achieve efficient KD in multiple acute leukaemia cell lines. To corroborate our findings, DNTTIP1‐KO cell lines were generated using the CRISPR/Cas9 system. Consistently, shRNA‐ or single‐guide RNA (sgRNA)‐mediated DNTTIP1 depletion significantly impaired in vitro proliferation across acute leukaemia cell lines, whereas it had no effect on the growth of chronic myeloid leukaemia‐derived K562 cells (Figures 2A,B and 2A‒G). Importantly, DNTTIP1 depletion did not affect the protein levels of other core MiDAC complex components, including MIDEAS, HDAC1 and HDAC2 (Figure S2A,B), indicating that the growth inhibitory effects are induced by DNTTIP1‐mediated global destabilisation or disruption of the MiDAC complex, without altering the expression of its other subunits. To extend these findings to clinically relevant models, we utilised primary patient samples and a B‐cell ALL PDX model, where siRNA‐mediated KD of DNTTIP1 similarly suppressed cell proliferation (Figure 2C,D). Building on these results, we further assessed the impact of DNTTIP1 on the self‐renewal and long‐term proliferative capacity of leukaemia cell lines through colony formation assays. Strikingly, DNTTIP1‐KD significantly diminished the colony‐forming capacity of these cells (Figures 2E and S2H,I). Morphological analysis of DNTTIP1‐depleted cells revealed hallmark apoptotic features, including cell shrinkage, nuclear fragmentation and cytoplasmic vacuolisation (Figure 2F). Flow cytometry analysis demonstrated that DNTTIP1 silencing induced G0/G1 phase arrest (Figures 2G and S2J), accompanied by a significant increase in apoptosis (Figure 2H). Concurrently, we observed a modest but consistent upregulation of differentiation markers (Figure S2K), suggesting DNTTIP1 may play a role in maintaining leukaemic cell stemness.

To determine DNTTIP1's role in leukaemogenesis, we introduced exogenous OE of DNTTIP1, which significantly enhanced cell proliferation (Figure 2I), increased colony‐forming capacity (Figure S2L) and reduced apoptosis (Figure S2M). These malignant phenotypes were reversible, with proliferative capacity being fully restored upon DNTTIP1 re‐expression in KD cells (Figure 2J). In vivo, we first established a human cell line‐derived xenograft model, observing that shRNA (shDNT_4)‐mediated DNTTIP1‐KD significantly suppressed leukaemia cells growth (Figure 2K,L) and prolonged survival (Figure 2M). To corroborate these findings, we silenced *Dnttip1* in murine models driven by AML1‐ETO (AE9a) or MLL‐AF9. Following transplantation into sublethally irradiated recipients, *Dnttip1*‐depleted cells exhibited markedly impaired leukaemogenic potential. Specifically, *Dnttip1*‐KD recipients exhibited stunted leukaemia progression (Figure 2N), characterised by a markedly reduced leukaemic burden in the BM, spleen and PB, as well as significantly improved survival relative to controls (Figure 2O‒T).

### 3.3 HDAC1/2 are critical for DNTTIP1‐mediated leukaemogenesis

**FIGURE 3:**
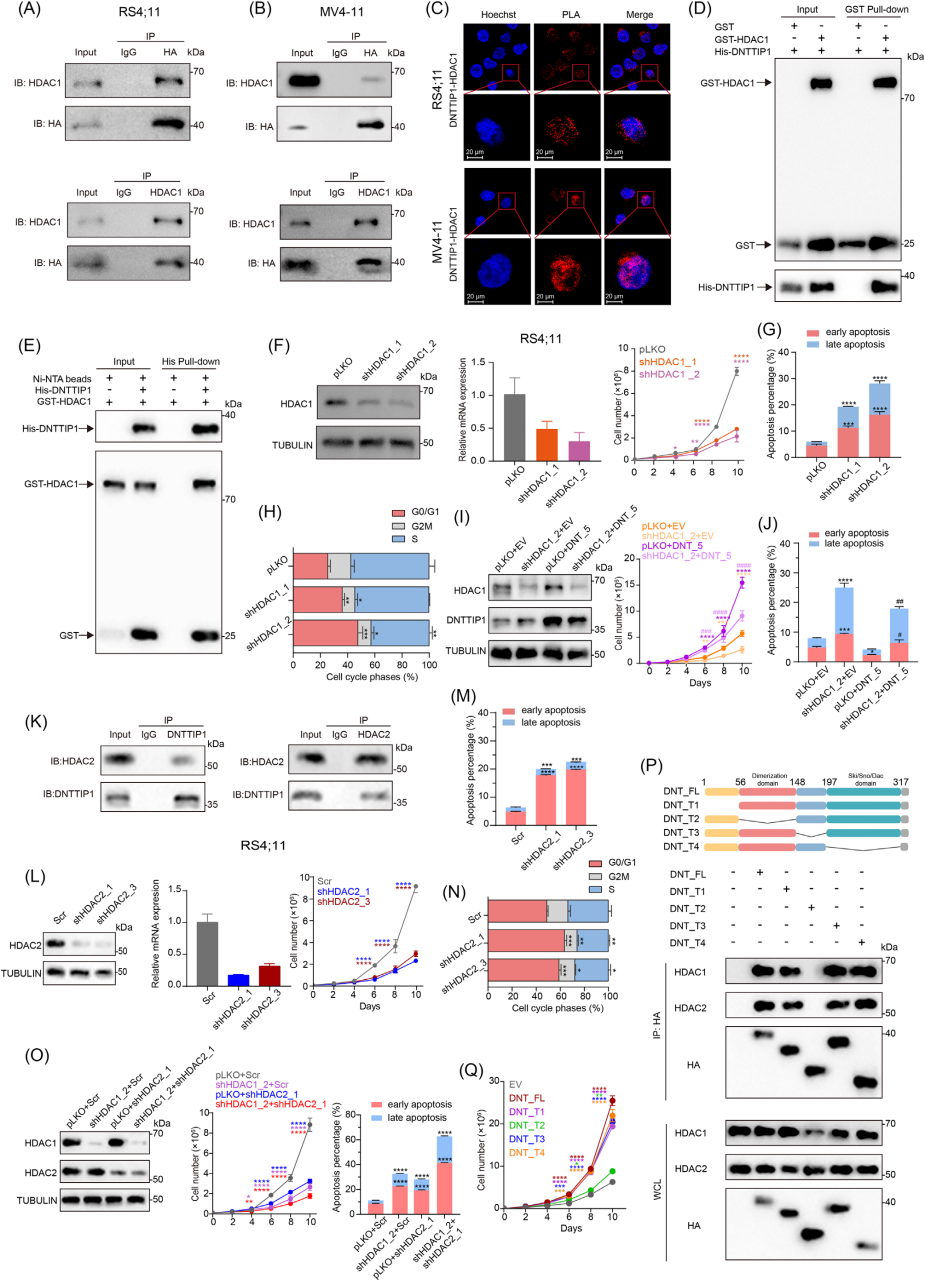
Histone deacetylase 1/2 (HDAC1/2) are critical for deoxynucleotidyl transferase terminal‐interacting protein 1 (DNTTIP1)‐mediated leukaemogenesis. (A and B) Co‐immunoprecipitation (Co‐IP) analysis in RS4;11 (A) and MV4;11 (B) cells stably expressing HA‐tagged DNTTIP1. Cell lysates were immunoprecipitated with anti‐HA (top) or anti‐HDAC1 (bottom) antibodies, followed by immunoblotting with the indicated antibodies. Input lanes represent 5% of total lysate (*n* = 3). (C) Proximity ligation assay (PLA, red) detecting endogenous DNTTIP1‒HDAC1 interaction in RS4;11 (top) and MV4‐11 (bottom) cells. Scale bar = 20 µM (*n* = 3). (D and E) GST/His‐tagged pull‐down assay showing the interaction between DNTTIP1 and HDAC1. (F) Immunoblot (left) and RT‐qPCR (middle) analyses demonstrating HDAC1 knockdown using two independent short hairpin RNAs (shRNAs) (shHDAC1_1 and shHDAC1_2). Cell counting of RS4;11 cells expressing shHDAC1_1 and shHDAC1_2 compared with control (pLKO) (right) (*n* = 3). (G) Flow cytometric analysis of Annexin V/propidium iodide (PI) staining in RS4;11 cells with HDAC1 knockdown (shHDAC1_1 and shHDAC1_2) versus control (pLKO) (*n* = 3). (H) Flow cytometric analysis was performed to assess cell cycle distribution in RS4;11 cells with HDAC1 knockdown (shHDAC1_1 and shHDAC1_2) versus control (pLKO) (*n* = 3). (I) Immunoblot analyses demonstrating the rescue effect of DNTTIP1 overexpression (DNTTIP1_5) upon HDAC1 knockdown (shHDAC1_2) cells (left). Cell counting analysis demonstrating that overexpression of DNTTIP1 (DNTTIP1_5) partially rescues the growth inhibition induced by HDAC1 knockdown (shHDAC1_2) in RS4;11 cells (middle). Quantification of early (Annexin V^+^ PI^−^) and late (Annexin V^+^ PI^+^) apoptotic populations in RS4;11 cells with DNTTIP1 overexpression on HDAC1 knockdown (*n* = 3). shHDAC1_2 + DNTTIP1_5 versus shHDAC1_2 + EV is denoted by symbol (#). All groups versus pLKO + EV is denoted by symbol (*). (K) Reciprocal Co‐IP analysis in RS4;11 cells demonstrated endogenous interaction between DNTTIP1 and HDAC2 (*n* = 3). (L) Immunoblot (left) and RT‐qPCR (middle) analyses demonstrating HDAC2 knockdown using two independent shRNAs (shHDAC2_1 and shHDAC2_3). Cell counting of RS4;11 cells expressing shHDAC2_1 and shHDAC2_3 compared with control (Scr) (right) (*n* = 3). (M) Flow cytometric analysis of Annexin V/PI staining in RS4;11 cells with HDAC2 knockdown (shHDAC2_1 and shHDAC2_3) versus control (Scr) (*n* = 3). (N) Flow cytometric analysis was performed to assess cell cycle distribution in RS4;11 cells with HDAC2 knockdown (shHDAC2_1 and shHDAC2_3) versus control (Scr) (*n* = 3). (O) Immunoblot analysis demonstrating the efficiency of HDAC1/2 double knockdown (left). Cell counting analysis showing that HDAC1/2 double knockdown inhibited proliferation more potently than individual knockdown of either HDAC1 or HDAC2 (middle). Quantification of early (Annexin V^+^ PI^−^) and late (Annexin V^+^ PI^+^) apoptotic populations in RS4;11 cells with HDAC1/2 double knockdown (*n* = 3). All groups versus pLKO + Scr is denoted by symbol (*). (P) Schematic diagram of DNTTIP1 domains (top). HEK‐293T cells were transiently transfected with HA‐tagged DNTTIP1 deletion constructs and cultured for 24 h. Western blot analysis was performed on total cell lysates and immunoprecipitated fractions using HA‐specific antibodies (bottom) (*n* = 3). (Q) Cell counting of RS4;11 cells transfected with HA‐tagged DNTTIP1 deletion constructs (*n* = 3).

As a core component of the MiDAC complex,^35^ DNTTIP1 may facilitate leukaemogenesis through HDAC1/2 recruitment. Reciprocal co‐immunoprecipitation (Co‐IP) assays in RS4;11 and MV4‐11 cells expressing HA‐tagged DNTTIP1 confirmed a physical interaction between DNTTIP1 and HDAC1 (Figure 3A,B), which was further validated by endogenous Co‐IP assays (Figure S3A). PLAs additionally demonstrated robust interaction between these proteins (Figure 3C), while GST pull‐down and His‐tagged pull‐down assays provided direct evidence of the DNTTIP1‒HDAC1 interaction (Figure 3D,E). Collectively, these complementary approaches support their functional cooperation in epigenetic reprogramming during leukaemogenesis.

To examine the functional contribution of HDAC1 in leukaemogenesis, we depleted HDAC1 in RS4;11 and MV4‐11 cells using shRNAs. HDAC1‐KD significantly impaired cell proliferation (Figures 3F and S3B) and triggered apoptosis (Figure 3G), accompanied by G0/G1 cell cycle arrest (Figure 3H). To elucidate the functional interplay between DNTTIP1 and HDAC1, we overexpressed DNTTIP1 in HDAC1‐deficient cells. This intervention significantly rescued the proliferation defects and reduced the elevated apoptosis rate observed in HDAC1‐KD cells (Figure 3I,J). However, given that DNTTIP1 can also interact with HDAC2 in addition to HDAC1, we extended our investigation to the DNTTIP1‒HDAC2 axis to delineate the comprehensive mechanism of DNTTIP1 in acute leukaemia. First, endogenous Co‐IP assays confirmed the physical interaction between DNTTIP1 and HDAC2 (Figure 3K). Consistently, HDAC2‐KD recapitulated the anti‐leukaemic phenotypes observed with HDAC1 depletion, including suppressed cell proliferation, enhanced apoptosis and G0/G1 cell cycle arrest (Figures 3L‒N and S3C). Notably, concurrent depletion of both HDAC1 and HDAC2 resulted in more profound anti‐leukaemic effects compared with single KD of either one (Figures 3O and S3D), suggesting that HDAC1 and HDAC2 may exert complementary and compensatory roles in mediating DNTTIP1‐driven leukaemogenesis. Pharmacological inhibition of HDAC1/2/3 using MS‐275 or CI‐994 also phenocopied these anti‐leukaemic effects (Figure S3E,F). To rule out the potential contribution of HDAC3 suppression to the observed anti‐leukaemic phenotypes, we treated acute leukaemia cells with highly selective HDAC1/2 inhibitors—Merck60 and low‐dose FK228 as well as a specific HDAC3 inhibitor RGFP966. Notably, Merck60 and FK228 exerted significant anti‐leukaemic effects, whereas RGFP966 showed minimal activity (Figure S3G,H). These findings collectively support that targeting HDAC1/2 may hold broad applicability for acute leukaemia therapeutic intervention, which is not dependent on HDAC3 inhibition. This is consistent with previous reports suggesting that HDAC3 inhibition may be effective only in leukaemias harbouring specific genetic backgrounds.^36^, ^37^

To further delineate the HDAC1/2‐interacting region within DNTTIP1, we conducted Co‐IP analyses in HEK293 cells transfected with HA‐tagged DNTTIP1 deletion constructs, which demonstrated that the DD is indispensable for mediating the direct physical association between DNTTIP1 and HDAC1/2 (Figure 3P), an observation that shares similarity with prior studies.^38^ In acute leukaemia cells expressing these DNTTIP1 deletion constructs, all truncations except Truncation 2 (T2, harbouring a deletion of the DD) recapitulated phenotypes nearly identical to those induced by full‐length DNTTIP1. T2, however, also exhibited weak but detectable phenotypes, albeit with a significantly reduced magnitude relative to the other truncations (Figure 3Q). To confirm that this interaction functionally contributes to leukaemogenesis, we treated cells with the HDAC1/2/3 inhibitor MS‐275. This treatment partially reversed the pro‐growth and anti‐apoptotic effects induced by DNTTIP1 OE (Figure S3I). Collectively, these findings confirm that the DD‐dependent DNTTIP1‒HDAC1/2 interaction is a key mediator of DNTTIP1's pro‐leukaemic activity, though non‐HDAC1/2‐mediated oncogenic functions of DNTTIP1 cannot be completely excluded.

### 3.4 DNTTIP1‒HDAC1/2‐mediated chromatin regulation modulates autophagy and apoptosis

**FIGURE 4:**
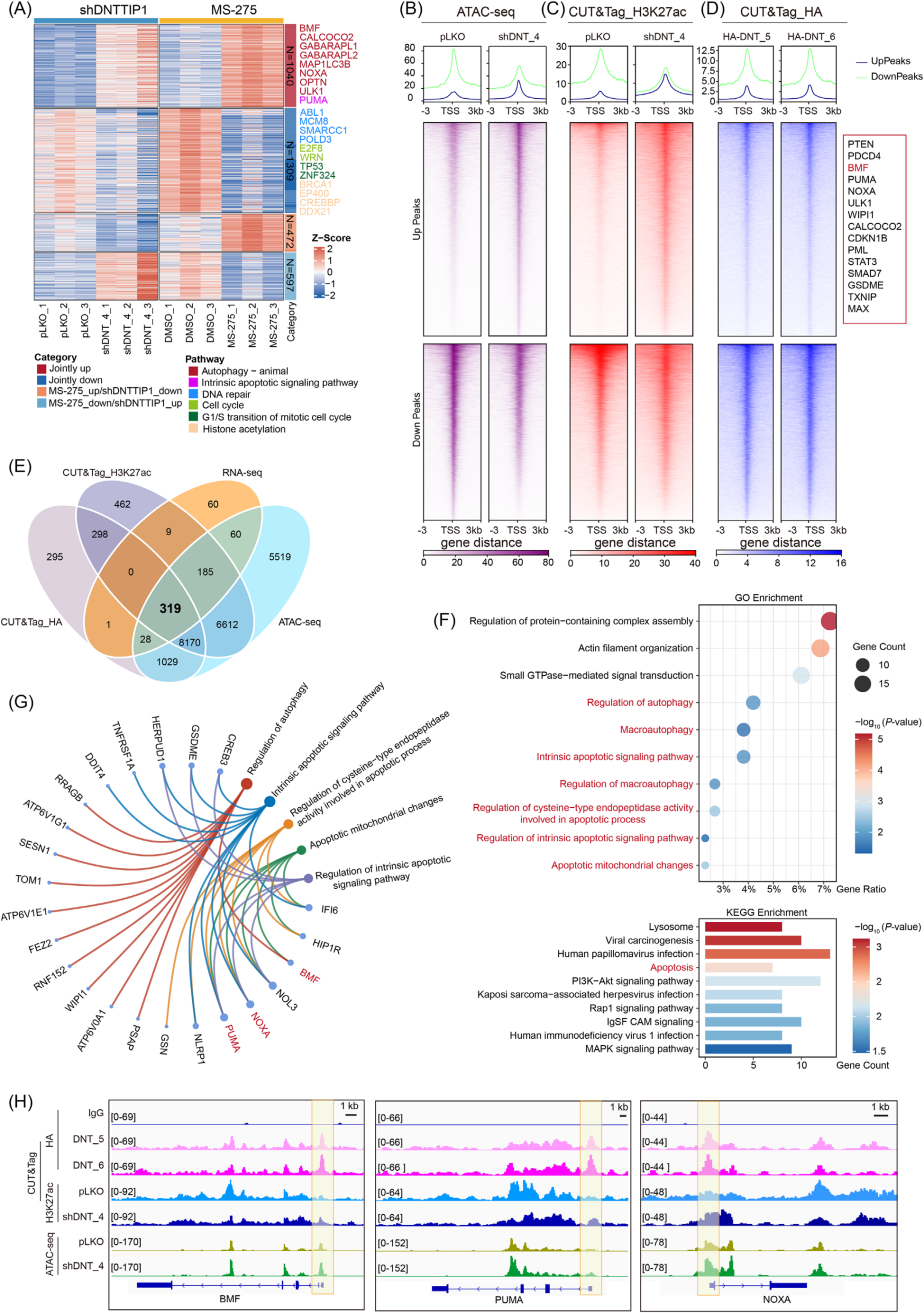
Deoxynucleotidyl transferase terminal‐interacting protein 1 (DNTTIP1)‒histone deacetylase 1/2 (HDAC1/2)‐mediated chromatin regulation modulates autophagy and apoptosis. (A) Heatmap illustrating the expression profiles of differentially expressed genes (DEGs) in RS4;11 cells following DNTTIP1 knockdown or MS‐275 treatment. (B‒D) Heatmap visualisation of coordinated changes in assay for transposase‐accessible chromatin using sequencing (ATAC‐seq) (B), H3K27ac (C) and HA‐DNTTIP1 (D) binding across responsive loci in RS4;11 leukaemia cells. (E) Venn diagram integrating multi‐omics data to delineate high‐confidence DNTTIP1 effector genes in RS4;11 cells. The intersection comprises four criteria: genes consistently upregulated across two RNA‐seq datasets, ATAC‐seq‐accessible loci upon DNTTIP1 knockdown (shDNT_4), H3K27ac‐targeted genes upon DNTTIP1 knockdown (shDNT_4) and direct DNTTIP1‐targeted genes. (F) Multi‐omics‐identified overlapping differentially expressed genes (DEGs) were visualised using bubble plots to show the top 10 Gene Ontology (GO) pathways (top) and bar plots to show the top 10 Kyoto Encyclopedia of Genes and Genomes (KEGG) pathways (bottom). (G) Chord diagrams illustrating the interconnected autophagy and apoptosis networks among multi‐omics overlapping genes. (H) Integrative genomic viewer (IGV) snapshot of cleavage under targets and tagmentation (CUT&Tag) (top: DNTTIP1; middle: H3K27ac) and ATAC‐seq (bottom) signals at the promoter regions of key BH3‐only genes (BCL2‐modifying factor [BMF], BCL2 binding component 3 [PUMA] and Phorbol‐12‐myristate‐13‐acetate‐induced protein 1 [NOXA]).

To comprehensively elucidate the oncogenic mechanisms of DNTTIP1‐mediated leukaemogenesis via HDAC1/2 axis, we integrated transcriptomic profiles of DNTTIP1‐depleted leukaemic cells and counterparts treated with MS‐275, an HDACi with activity against HDAC1/2/3, to identify convergent molecular vulnerabilities. Comparative RNA‐seq analysis revealed substantial transcriptome remodeling under both experimental conditions. Hierarchical clustering demonstrated both condition‐specific gene expression profiles and a core set of co‐regulated transcripts (Figure 4A). Specifically, we identified a set of overlapping differentially expressed genes (DEGs) between DNTTIP1‐depleted and MS‐275‐treated leukaemic cells (Figure S4A,B). This overlap suggests that a portion of DNTTIP1's pro‐leukaemic program is HDAC dependent, while also implying the existence of HDAC‐independent functions. Functional enrichment analysis of DEGs following DNTTIP1‐KD revealed perturbation‐specific pathway alterations, leveraging Gene Ontology (GO) and Kyoto Encyclopedia of Genes and Genomes (KEGG) annotations (Figure S4C,D). Prior studies have established a role for DNTTIP1 in cell cycle regulation and DNA repair,^21^, ^22^ a finding consistent with the pathway enrichment results of our downregulated DEGs. Notably, our analysis also identified previously unreported pathways associated with upregulated DEGs, which may represent novel molecular events implicated in DNTTIP1‐mediated leukaemogenesis.

Given the established DNTTIP1‒HDAC1/2 interaction, we hypothesised that DNTTIP1 modulates chromatin state. Assay for transposase‐accessible chromatin using sequencing (ATAC‐seq) analysis revealed that DNTTIP1‐KD markedly increased global chromatin accessibility (Figure 4B), suggesting its role in maintaining chromatin compaction. To mechanistically connect these changes to DNTTIP1‒HDAC1/2‐mediated transcriptional regulation, we performed H3K27 acetylation (H3K27ac)‐targeted cleavage under targets and tagmentation (CUT&Tag) sequencing. Strikingly, DNTTIP1‐KD induced a genome‐wide surge in H3K27ac levels, with pronounced binding signals near transcriptional start sites (TSS) (Figure 4C). DNTTIP1‐KD increased chromatin accessibility and enhanced H3K27ac enrichment at promoter regions (Figure S4E,F). These results firmly establish DNTTIP1 as a pivotal regulator of chromatin architecture, functioning through the precise spatiotemporal recruitment of HDAC1 to specific genomic loci. To elucidate the direct targets of DNTTIP1, we performed CUT&Tag analysis in two single‐clone cells stably overexpressing HA‐tagged DNTTIP1, which displayed pronounced DNTTIP1 occupancy near TSS enriched with enhanced H3K27ac and chromatin accessibility (Figure 4D). Moreover, the two single‐clone cells displayed highly concordant DNTTIP1 binding profiles, with substantial overlap in target genes (Figure S4G,H), underscoring the specificity and reproducibility of its genomic localisation. We performed enrichment analysis on genes corresponding to the overlapping peaks identified from CUT&Tag (Figure S4I), revealing significant enrichment in cancer‐related pathways, including apoptosis, autophagy and p53 signalling pathway. To pinpoint the most critical DNTTIP1‒HDAC1/2‐regulated genes, we integrated multi‐omics data from RNA‐seq, ATAC‐seq and two independent CUT&Tag (HA‐targeted DNTTIP1 and H3K27ac upon DNTTIP1‐KD), identifying 319 overlapping genes (Figure 4E). GO and KEGG enrichment analysis of these genes showed specific enrichment in autophagy and apoptosis pathways (Figure 4F), with significant contribution from BH3‐only family members. Notably, our multi‐omics analysis revealed BMF as the sole BH3‐only gene involved in both autophagy and apoptosis, distinguishing it from Phorbol‐12‐myristate‐13‐acetate‐induced protein 1 (NOXA) and BCL2 binding component 3 (PUMA) (Figure 4G). This positions BMF as a key downstream effector of DNTTIP1‐mediated transcriptional regulation. Comparative genomic profiling of the three overlapping BH3‐only genes revealed that BMF exhibited the most pronounced epigenetic activation signature, characterised by significantly increased chromatin accessibility and strong enrichment of both DNTTIP1 and H3K27ac at its promoter regions, surpassing PUMA and NOXA, which showed only modest changes primarily within their promoter regions (Figure 4H).

### 3.5 DNTTIP1 interacts with SP1 to repress the transcription of BMF

**FIGURE 5:**
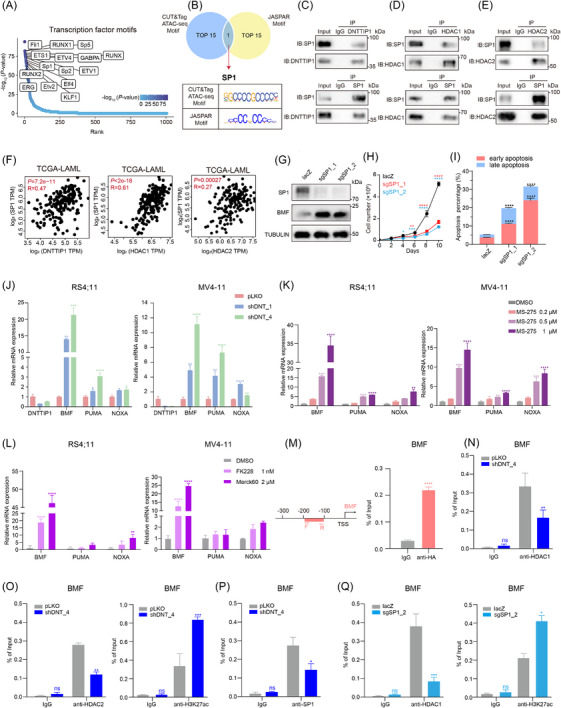
Deoxynucleotidyl transferase terminal‐interacting protein 1 (DNTTIP1) interacts with SP1 to repress the transcription of BCL2‐modifying factor (BMF). (A) Motif analysis identifies the top 15 transcription factors enriched within DNTTIP1‐bound genomic regions. (B) Venn diagram showing the overlap of multi‐omics binding motifs (assay for transposase‐accessible chromatin using sequencing [ATAC‐seq] and cleavage under targets and tagmentation [CUT&Tag]) and predicated JASPAR motifs anchored to the DNTTIP1‐bound BMF promoter in RS4;11 cells (left). Table presenting the SP1 motif logo and position‐weight matrix (right). (C‒E) Reciprocal co‐immunoprecipitation (Co‐IP) analysis in RS4;11 cells demonstrated binding of endogenous SP1 to DNTTIP1 and HDAC1/2 (*n* = 3). (F) Correlation analysis of SP1 with DNTTIP1 and HDAC1/2 in The Cancer Genome Atlas (TCGA)‐LAML. Pearson correlation coefficients were computed using GEPIA 2 (http://gepia2.cancer‐pku.cn/#index). (G) Immunoblot (left) and RT‐qPCR (middle) analyses demonstrating SP1 knockout using two different single‐guide RNAs (sgSP1_1 and sgSP1_2). (H) Cell counting of RS4;11 cells expressing sgSP1_1 and sgSP1_2 compared with control (lacZ) (right) (*n* = 3). (I) Flow cytometric analysis of Annexin V/propidium iodide (PI) staining in RS4;11 cells with SP1 knockout (sgSP1_1 and sgSP1_2) versus control (lacZ) (*n* = 3). (J‒L) RT‐qPCR analysis of mRNA expression of BH3‐only genes (BMF, PUMA and NOXA) in leukaemia cells following DNTTIP1 knockdown (J), MS‐275 treatment (K) or FK228/Merck60 treatment (L). Data represent mean ± standard deviation (SD) of three independent experiment. ^*^ *p *< .05, ^**^ *p *< .01, ^***^ *p *< .001, ^****^ *p *< .0001. (M) Schematic of chromatin immunoprecipitation quantitative PCR (ChIP‐qPCR) locus‐specific BMF primer locations (left). ChIP‐qPCR analysis showing the HA (DNTTIP1) enrichment on BMF promoter in HA‐tagged DNTTIP1 overexpressed RS4;11 cells (right). (N) ChIP‐qPCR analysis showing the HDAC1 enrichment at BMF promoter in control (pLKO) and DNTTIP1 knockdown (shDNT_4) RS4;11 cells. (O) ChIP‐qPCR analysis showing the HDAC2 (left) and H3K27ac (right) enrichment at BMF promoter in control (pLKO) and DNTTIP1 knockdown (shDNT_4) RS4;11 cells. (P) ChIP‐qPCR analysis showing the SP1 enrichment at BMF promoter in control (pLKO) and DNTTIP1 knockdown (shDNT_4) RS4;11 cells. (Q) ChIP‐qPCR analysis showing the HDAC1 (left) and H3K27ac (right) enrichment at BMF promoter in control (lacZ) and SP1 knockout (sgSP1_2) RS4;11 cells.

To identify transcription factors (TFs) that cooperate with DNTTIP1 in regulating BMF transcription, we performed motif enrichment analysis on peaks shared between ATAC‐seq and CUT&Tag data. Ranking the top 15 enriched motifs revealed potential co‐regulators, including the leukaemia‐associated factors RUNX1 and SP1, suggesting their potential cooperation with DNTTIP1 (Figure 5A). To increase specificity, we refined our analysis by anchoring to DNTTIP1‐bound sequences at the BMF promoter (DNTTIP1‐targeted CUT&Tag) and predicting motifs using the JASPAR database. Integrating the top 15 motifs from ATAC‐seq, CUT&Tag and JASPAR predictions revealed SP1 as the only candidate TF common to all datasets (Figure 5B). Notably, SP1 is a known HDAC1 and HDAC2 interactor, suggesting it may bridge DNTTIP1 to HDAC1/2‐dependent epigenetic silencing at the BMF promoter. The exclusivity of SP1 in our motif analysis highlights the specificity of DNTTIP1‒SP1 cooperation and aligns with SP1's established role as a master regulator of haematopoietic transcription.

To validate this interaction, endogenous Co‐IP assays confirmed the physical interactions of SP1 with DNTTIP1, HDAC1 and HDAC2 in leukaemia cells (Figure 5C‒E). Consistent with this molecular interaction, SP1 expression positively correlated with these proteins in AML patient datasets (Figure 5F), underscoring the clinical relevance of this regulatory axis. To further explore whether SP1 modulates BMF transcriptional activity downstream of the DNTTIP1‒HDAC1/2 axis, we analysed BMF expression in SP1‐KO leukaemia cells and found a significant upregulation of BMF, confirming SP1's role in repressing BMF transcription (Figure 5G). Furthermore, SP1‐KO significantly inhibited leukaemic cell proliferation and promoted apoptosis (Figure 5H,I), which aligns with the pro‐leukaemic function of the DNTTIP1‒HDAC1/2 axis.

To validate our hypothesis from multi‐omics data, we assessed the expression of three BH3‐only genes across diverse acute leukaemia cell lines. Intriguingly, DNTTIP1‐KD or MS‐275 treatment consistently upregulated all three genes (Figures 5J,K and S5A,B), with BMF showing significantly stronger induction than PUMA and NOXA. Consistently, treatment with the highly selective HDAC1/2 inhibitor Merck60 or low‐dose FK228, which also targets HDAC1/2, yielded comparable results (Figure 5L), further confirming that inhibition of the DNTTIP1‒HDAC1/2 axis modulates the expression of BH3‐only genes. In contrast, DNTTIP1 OE suppressed these BH3‐only genes (Figure S5C), while DNTTIP1 lacking the HDAC1/2‐interacting DD failed to recapitulate the suppressive effect of the full‐length protein (Figure S5D), further supporting our model of DNTTIP1‒HDAC1/2‐mediated transcriptional regulation of BH3‐only genes. ChIP‐qPCR analysis revealed specific enrichment of DNTTIP1 at the BMF promoter region in acute leukaemia cells (Figure 5M). Furthermore, DNTTIP1‐KD reduced HDAC1 and HDAC2 recruitment while increasing H3K27ac enrichment at the BMF promoter (Figure 5N,O). We further quantified SP1 enrichment at the BMF promoter region and found a significant reduction in SP1 occupancy there in DNTTIP1‐silenced leukaemia cells (Figure 5P). Consistently, CRISPR/Cas9‐mediated SP1 ablation reproduced the epigenetic changes from DNTTIP1 silencing, specifically reducing HDAC1 recruitment and enhancing H3K27ac enrichment at the BMF promoter (Figure 5Q). Together, these data strongly support a model wherein DNTTIP1 interacts with SP1 and HDAC1/2 to orchestrate the transcriptional repression of BMF in acute leukaemia cells through modulating histone acetylation at its promoter region.

### 3.6 DNTTIP1‒HDAC1/2‒BMF axis drives acute leukaemia progression by suppressing apoptosis and autophagy

**FIGURE 6:**
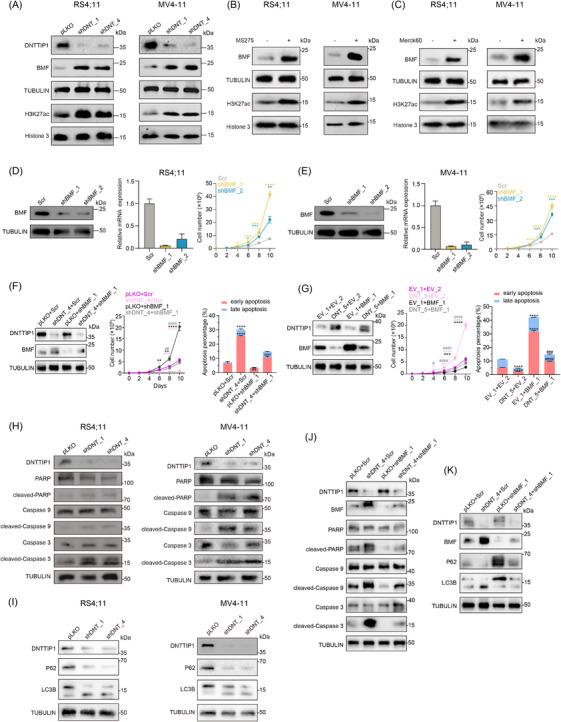
Deoxynucleotidyl transferase terminal‐interacting protein 1 (DNTTIP1)‒histone deacetylase 1/2 (HDAC1/2)‒BCL2‐modifying factor (BMF) axis drives acute leukaemia progression by suppressing apoptosis and autophagy. (A) Levels of BMF and H3K27ac detected by Western blotting after knockdown of DNTTIP1 in RS4;11 cells and MV4‐11 cells. (B and C) Levels of BMF and H3K27ac detected by Western blotting following treatment with MS‐275 (B) or Merck60 (C) in RS4;11 cells and MV4‐11 cells. (D and E) Immunoblot (left) and RT‐qPCR (middle) analyses demonstrating BMF knockdown using two independent short hairpin RNAs (shRNAs) (shBMF_1 and shBMF_2). Cell counting of RS4;11 and MV4‐11 cells expressing shBMF_1 and shBMF_2 compared with control (scramble) (right) (*n* = 3). (F) Immunoblot analyses demonstrating the rescue effect of BMF knockdown (shBMF_1) upon DNTTIP1 knockdown (shDNT_4) (left). Cell counting of DNTTIP1‐deficient RS4;11 cells with BMF knockdown (middle). Quantification of early (Annexin V^+^ PI^−^) and late (Annexin V^+^ PI^+^) apoptotic populations in DNTTIP1‐deficient RS4;11 cells with BMF knockdown (right) (*n* = 3). shDNT_4 + shBMF_1 versus shDNT_4 + scramble is denoted by symbol (#). All groups versus pLKO + scramble is denoted by symbol (*). ^#^ *p *< .05, ^##^ *p *< .01, ^###^ *p *< .001, ^####^ *p *< .0001, ^*^ *p *< .05, ^**^ *p *< .01, ^***^ *p *< .001, ^****^ *p *< .0001. (G) Immunoblot analyses demonstrating the rescue effect of BMF overexpression (BMF_1) upon DNTTIP1 overexpression (DNT_5) (left). Cell counting of RS4;11 cells with DNTTIP1 overexpression and concurrent BMF overexpression (middle). Quantification of early (Annexin V^+^ PI^−^) and late (Annexin V^+^ PI^+^) apoptotic populations in RS4;11 cells with concurrent DNTTIP1 and BMF overexpression (right) (*n* = 3). DNT_5 + BMF_1 versus DNT_5 + EV_2 is denoted by symbol (#). All groups versus EV_1 + EV_2 denoted by symbol (*). ^#^ *p *< .05, ^##^ *p *< .01, ^###^ *p *< .001, ^####^ *p *< .0001, ^*^ *p *< .05, ^**^ *p *< .01, ^***^ *p *< .001, ^****^ *p *< .0001. (H and I) Western blot analysis of the apoptosis‐related (H) and autophagy‐related (I) protein levels after DNTTIP1 knockdown in RS4;11 cells and MV4‐11 cells. (J and K) Western blot analysis showing the apoptosis‐related (J) and autophagy‐related (K) protein changes in DNTTIP1‐deficient RS4;11 cells with BMF knockdown.

DNTTIP1 depletion elevated both BMF protein and global H3K27ac levels in acute leukaemia cells (Figure 6A). Similarly, MS‐275 or the selective HDAC1/2 inhibitor Merck60 treatment recapitulated these effects, increasing BMF and H3K27ac level (Figure 6B,C). To elucidate the role of the DNTTIP1‒HDAC1/2‒BMF axis in leukaemogenesis, we performed BMF‐KD, which increased leukaemia cell proliferation (Figure 6D,E), supporting BMF as a critical tumour suppressor. Rescue experiments revealed that BMF depletion partially reversed the anti‐proliferative and pro‐apoptotic effects of DNTTIP1‐KD in acute leukaemia cells (Figure 6F), whereas BMF OE partially mitigated the pro‐proliferative and anti‐apoptotic effects of ectopic DNTTIP1 expression in these cells (Figure 6G). Mechanistically, genetic ablation of DNTTIP1 induced concomitant activation of both apoptotic and autophagic pathways (Figure 6H,I). BMF depletion markedly abrogated these dual effects (Figure 6J,K), unequivocally establishing BMF as the pivotal downstream effector of DNTTIP1‐dependent cell fate regulation.

Our data support a model wherein DNTTIP1 maintains leukaemic cell survival by recruiting HDAC1/2 within the context of MiDAC to epigenetically repress BMF. Genetic or pharmacological disruption of this axis activates complementary cell death mechanisms, suggesting that targeting DNTTIP1 may potentiate existing leukaemia therapies.

### 3.7 DNTTIP1 loss enhances chemosensitivity, and HDACi/BCL2i/poly(ADP‐ribose) polymerase inhibitor triple therapy shows potent synergy in acute leukaemia

**FIGURE 7:**
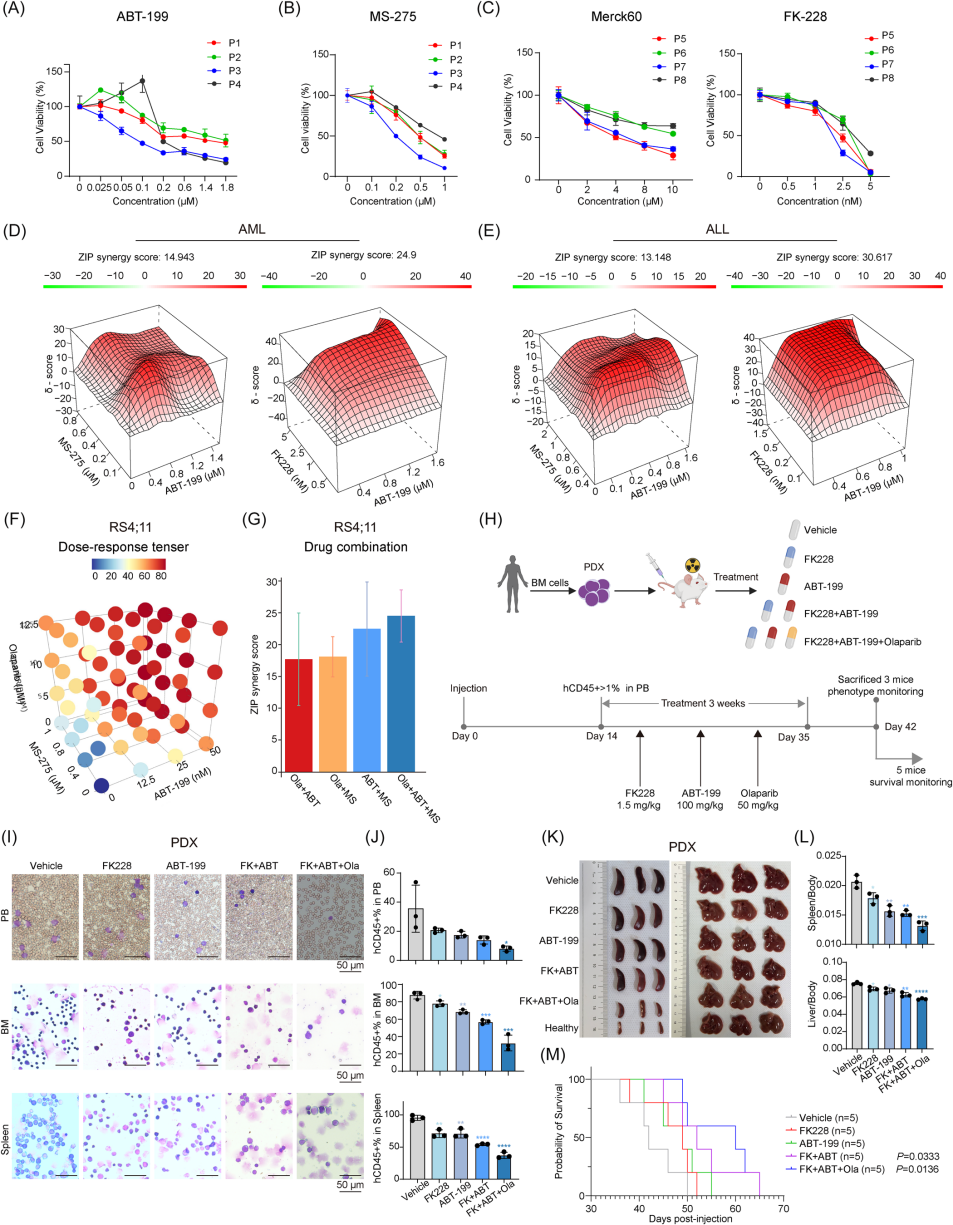
Deoxynucleotidyl transferase terminal‐interacting protein 1 (DNTTIP1) loss enhances chemosensitivity and histone deacetylase inhibitors (HDACi)/BCL2 inhibitor (BCL2i)/poly(ADP‐ribose) polymerase inhibitor (PARPi) triple therapy shows potent synergy in acute leukaemia. (A and B) Viability of primary bone marrow mononuclear cells (BMNCs) from three acute myeloid leukaemia (AML) patients and one acute lymphoblastic leukaemia (ALL) patient was assessed using the CTG assay following in vitro treatment with ABT‐199 (A) or MS‐275 (B) at a range of concentrations for 48 h. (C) Viability of primary BMNCs from three additional AML patients and one ALL patient was assessed using the CTG assay following in vitro treatment with Merck60 (left) or FK228 (right) at a range of concentrations for 48 h. (D and E) Primary BMNCs from AML (D) and ALL (E) patients were treated with ABT‐199 in combination with either MS‐275 or FK228 at multiple concentrations for 48 h. (F) RS4;11 cells were exposed to escalating doses of ABT‐199, MS‐275, Olaparib, or their combinations for 48 h. Cell viability was assessed using the CTG assay, and drug interaction landscapes were generated to evaluate combinatorial effects. (G) Bar charts showing ZIP synergy scores for pairwise combinations (Olaparib + ABT‐199, Olaparib + MS‐275, ABT‐199 + MS‐275) and the triple combination (Olaparib + ABT‐199 + MS‐275). (H) Schematic overview of the experimental workflow using an ALL patient‐derived xenograft (PDX) mouse model. Two weeks following cell inoculation, mice were randomly allocated into five groups and treated with vehicle, FK228 (1.5 mg/kg, twice weekly), ABT‐199 (100 mg/kg, daily), FK228 combined with ABT‐199, or FK228 plus ABT‐199 and Olaparib (50 mg/kg, daily) for three consecutive weeks. Six weeks after cell inoculation, three mice per group were sacrificed for phenotypic analysis, while the remaining five mice in each group were monitored for survival. (I) Giemsa staining of peripheral blood (PB) smears, BMNCs and spleen mononuclear cells from mice in each group. (J) Percentage of hCD45^+^ leukaemic blasts in the PB, bone marrow (BM) and spleen of recipient mice in each group (*n* = 3 per group). (K) Representative images of spleen and liver sizes in recipient mice from each group (*n* = 3 per group). (L) Spleen and liver weights relative to body weight in recipient mice from each group (*n* = 3 per group). (M) Kaplan‒Meier survival plots were employed to illustrate the survival of mice, with curve comparisons analysed using the log‐rank test (*n* = 5 per group).

Given the potential of DNTTIP1 as an anti‐leukaemic target, we sought to assess its clinical relevance and therapeutic value in the context of acute leukaemia. Genetic depletion of DNTTIP1 significantly sensitised leukaemic cells to frontline chemotherapeutic agents, including Ara‐C, DNR, VCR and DOX (Figure S6A,B). Additionally, DNTTIP1‐KD sensitised leukaemic cells to the HDAC1/2/3 inhibitor MS‐275, triggering heightened apoptosis (Figure S6C), indicating that DNTTIP1 may modulate the function of HDACs in acute leukaemia. Next, we performed pharmacological interventions in leukaemia cells using the BH3 mimetic ABT‐199 with MS‐275. Both monotherapies effectively inhibited proliferation and promoted apoptosis in acute leukaemia cells, with the combination therapy yielding even greater efficacy (Figure S6D,E). To further validate the therapeutic potential of the drugs in acute leukaemia, we conducted in vitro experiments using bone marrow mononuclear cells (BMNCs) derived from AML or ALL patients and assessed viability using the CellTiter‐Glo (CTG) assay. While drug sensitivity varied slightly between patient samples and cell lines, ABT‐199 and a panel of HDACi including MS‐275, CI‐994, Merck60 and FK228 potently inhibited leukaemic cell proliferation in both AML and ALL patient‐derived BMNCs (Figures 7A‒C and S6F). In contrast, treatment with the selective HDAC3 inhibitor RGFP966 exerted minimal anti‐proliferative effects on patient‐derived leukaemic BMNCs in vitro, even at high concentrations (Figure S6G). This finding aligns with our previous observations in the RS4;11 cell line model, where RGFP966 failed to induce significant leukaemic cell apoptosis compared to HDAC1/2‐targeting inhibitors. This may indicate that HDAC1/2 may play a more prominent role than HDAC3 in mediating the anti‐leukaemic activity of HDACi, highlighting the therapeutic relevance of targeting the DNTTIP1‒HDAC1/2 axis in acute leukaemia.

Building on these findings, we investigated combination therapy strategies in leukaemia cells, with a focus on combining FDA‐ or National Medical Products Administration (NMPA)‐approved HDACi including FK228 and MS‐275 that could target HDAC1 and HDAC2 with the FDA‐approved BH3 mimetic ABT‐199, a strategy that holds enhanced potential for subsequent clinical translation. In parallel, the therapeutic efficacy of a recently established AML regimen, 5‐AzaC combined with ABT‐199, was also evaluated in ALL cells. Both combination strategies exhibited a significant synergistic inhibitory effect on ALL cell proliferation. However, the MS‐275 and ABT‐199 combination demonstrated superior synergy compared to the 5‐AzaC and ABT‐199 regimen, as quantified by a significantly higher ZIP synergy score (Figure S6H). Importantly, the combination therapy regimen of MS‐275 or FK228 in combination with ABT‐199 also exerted potent synergistic cytotoxic effects in AML and ALL patient primary BMNCs (Figure 7D,E), underscoring its translational potential. Since DNTTIP1‐KD suppressed DNA repair pathways (Figure S4C), we hypothesised that its depletion would exacerbate genomic instability in leukaemia cells, thereby sensitising them to poly(ADP‐ribose) polymerase (PARP) inhibitors (PARPi)**—**a class of therapeutics that selectively target DNA damage repair deficiencies. Alkaline comet assays in PARPi (Olaparib)‐treated cells revealed markedly longer tails and elevated tail DNA in DNTTIP1‐KD cells (Figure S6I,J). These findings demonstrate that DNTTIP1 loss compromises genomic integrity and sensitises leukaemia cells to PARP inhibition. Next, we evaluated whether combinatorial targeting of DNA repair could enhance therapeutic efficacy. Strikingly, the triple combination of Olaparib (PARPi), ABT‐199 (BH3 mimetic) and MS‐275 (HDACi) exhibited significantly stronger synergy than any dual‐drug regimen (Figure 7F,G), suggesting a potential synthetic lethal interaction that could be exploited therapeutically in acute leukaemia. Next, we assessed the efficacy of this triple‐combination therapy, comprising FK228, ABT‐199 and Olaparib, in an acute leukaemia PDX model (Figure 7H). Recipient mice treated with the triple combination exhibited reduced infiltration of human leukaemic cells in the PB, BM and spleen compared with control mice (Figure 7I,J). Notably, the triple combination resulted in less severe splenomegaly and hepatomegaly than monotherapy or dual‐drug combinations (Figure 7K,L) and significantly prolonged the overall survival of PDX‐bearing mice (Figure 7M). Furthermore, the triple combination did not induce overt toxicity, as evidenced by the lack of significant body weight changes among mice in all treatment groups (Figure S6K). Collectively, this triple combination shows potent anti‐leukaemic activity and favourable safety in acute leukaemia PDX model.

## 4 DISCUSSION

Despite substantial improvements in acute leukaemia survival outcomes over the past five decades, the fundamental framework of induction chemotherapy has remained largely unaltered.^39^, ^40^ While genomic studies have enabled novel therapies,^41^, ^42^ genetic analysis alone is insufficient to identify all critical pathogenic mechanisms, highlighting the growing importance of epigenetic dysregulation. Our study demonstrates that DNTTIP1 recruits HDAC1/2 to chromatin, promoting histone deacetylation and chromatin compaction. Through the DNTTIP1‒HDAC1/2‒BMF axis, this epigenetic silencing mechanism coordinately regulates autophagy and apoptosis to drive leukaemogenesis. Notably, both genetic disruption and pharmacological inhibition of this axis potently suppress leukaemic progression, revealing its therapeutic relevance.

The intricate equilibrium between histone acetylation and deacetylation represents a fundamental epigenetic regulatory mechanism controlling chromatin architecture. In human malignancies, recurrent HDAC dysregulation perturbs this delicate balance, driving malignant transformation,^16^ with aberrant activity of class I HDACs being particularly implicated in the pathogenesis of various cancers.^43^ Our analysis revealed that HDAC1 is closely involved in acute leukaemia among HDAC family members, which is consistent with prior studies demonstrating that elevated HDAC1 expression correlates with poor prognosis in leukaemia patients.^10^ Comprehensive profiling of HDAC1 interactors in acute leukaemia unexpectedly identified the understudied DNTTIP1 as its most prominent and specific binding partner. Notably, DNTTIP1 interacts selectively with HDAC1 and HDAC2 to the exclusion of other HDAC family members. This interaction exhibits particularly high affinity, surpassing even well‐characterised components of canonical HDAC complexes. This finding aligns with the emerging view that DNTTIP1 orchestrates cancer progression through context‐dependent molecular partnerships. In nasopharyngeal carcinoma, DNTTIP1 recruits HDAC1 to the DUSP2 locus, activating pro‐metastatic ERK signaling pathways.^24^ The same DNTTIP1‒HDAC1 interaction drives oral carcinogenesis by mediating p53 deacetylation. In lung cancer, DNTTIP1 exhibits dual functionality—it engages HDAC1 to suppress p53 activity^23^ and recruits LSD1 to initiate epithelial‒mesenchymal transition through E‐cadherin repression. Although its role in haematologic malignancies was previously limited to computational predictions in AML, our work now definitively links DNTTIP1 to acute leukaemia pathogenesis and clinical outcomes.

Integrative bioinformatics analyses of diverse acute leukaemia datasets revealed DNTTIP1 OE as a recurrent feature with clinical relevance. Functional validation revealed that DNTTIP1 sustains leukaemic cell proliferation through epigenetic reprogramming, with its genetic inhibition eliciting profound anti‐leukaemic responses. Earlier studies have shown that the MiDAC complex has exceptionally high catalytic activity towards nucleosome substrates in deacetylating the histone H3 tail,^18^, ^44^ implying the potential epigenetic function of DNTTIP1. Our results reveal that DNTTIP1 depletion impairs HDAC1/2 recruitment to chromatin, leading to increased H3K27ac deposition at the BMF promoter. This epigenetic remodeling activates both autophagy and apoptosis pathways via the DNTTIP1‒HDAC1/2‒BMF axis, ultimately inhibiting leukaemia progression. Motif analysis reveals that DNTTIP1 may cooperate with SP1 to form a transcriptional regulatory complex to regulate BMF expression, potentially coordinating autophagy and apoptosis pathways. Our study validates the DNTTIP1‒SP1 interaction and elucidates its regulatory impact on BMF expression. This finding aligns with SP1's established role in modulating these cellular pathways in leukaemia,^45^ further supporting its potential as a co‐regulator of DNTTIP1.

BMF functions as a pivotal mediator of both autophagy and apoptosis pathways, coordinately regulating these vital cellular processes in acute leukaemia. In unstressed cells, BMF is tethered to cytoskeletal structures through dynein light chain 2 (DLC2) binding, but stress‐induced liberation enables its mitochondrial translocation and engagement with pro‐survival BCL2 family proteins.^26^, ^46^ The BCL2 family is demarcated by distinct BH domains: pro‐survival proteins typically contain all four BH domains (BH1‒4), and pro‐apoptotic proteins, including multi‐domain proteins (with BH1‒4) and BH3‐only actors such as BMF.^27^ Notably, Beclin1, which is a key regulator in the initiation of autophagy, contains a BH3 domain that facilitates its interaction with BCL2, enabling BCL2‐mediated sequestration and inhibition of autophagy initiation.^28^, ^47^ Our data demonstrate that DNTTIP1 suppression transcriptionally upregulates BMF, concurrently activating apoptosis and autophagy in leukaemic cells. This dual effect likely results from BMF‐mediated disruption of BCL2 complexes with BAX/BAK and Beclin‐1,^48^ consistent with reports of BH3‐only proteins dually activating autophagy and apoptosis.^29^ In contrast, conflicting reports indicate that BMF stabilises the inhibitory Beclin‐1/BCL2 interaction and couples the autophagy complex to myosin V, thereby suppressing autophagosome formation in adherent cells.^49^, ^50^ However, as a BH3‐only protein, BMF can also competitively bind to pro‐survival BCL2 family members, liberating Beclin‐1 from its inhibitory complexes and initiating autophagosome formation.^29^ Our findings demonstrate that elevated BMF expression induces autophagy activation in acute leukaemia cells. However, further experimental validation is required to confirm these mechanistic insights. In this study, we confirmed that BMF functions as a critical dual regulator of apoptosis and autophagy in acute leukaemia, functioning as a tumour suppressor. These findings establish BMF as a tumour‐suppressive dual regulator in acute leukaemia, expanding its validated anti‐cancer role across malignancies^26^, ^51^ and advancing insights into cell death regulation in haematologic cancers.

Therapeutic targeting of the DNTTIP1‒HDAC1/2‒BMF axis represents a promising strategy for acute leukaemia. To evaluate the potential of this approach, we first assessed the activity of multiple HDACi against acute leukaemia cells. Our findings indicate that inhibitors encompassing HDAC1 and HDAC2 within their spectrum of activity exhibit broad efficacy, suggesting that targeting these enzymes is sufficient to elicit a robust anti‐leukaemic response. However, the contribution of other HDAC subtypes cannot be excluded, particularly given the growing evidence that additional class I HDACs, including HDAC3 and HDAC8, also play critical roles in tumourigenesis.^52^, ^53^ This complexity underscores that the therapeutic potential of HDACi in acute leukaemia remains incompletely defined and is likely context dependent. Indeed, emerging evidence highlights the diverse therapeutic potential of HDACi in leukaemia, with distinct agents demonstrating efficacy against specific subtypes. Specifically, MS‐275 treatment demonstrates efficacy in AML1‐ETO‐positive AML^54^; novel dual HDAC/MLL1 inhibitors exhibit enhanced anti‐leukaemic activity in MLL‐rearranged cells^55^; and the HDAC10 inhibitor PZ48 has shown promise against ALL cells in a Danio rerio model.^56^ Four HDACi have received FDA approval for haematologic malignancies, including Vorinostat, Belinostat, Panobinostat and FK228.^6^ In China, NMPA has approved Chidamide, and MS‐275 was additionally licensed in 2024,^57^ reflecting the rapidly evolving clinical acceptance of this drug class.

ABT‐199, the only FDA‐approved BCL2 antagonist, has revolutionised the treatment landscape for AML and high‐risk myelodysplastic syndrome, particularly when combined with DNA hypomethylating agents in unfit or elderly AML patients.^58^ In contrast, our identification of the DNTTIP1‒HDAC1/2‒BMF axis as a critical target in acute leukaemia provides a strong rationale for combining HDACi with BH3 mimetics to achieve synergistic anti‐leukaemic activity. Consistent with this concept, our study found that combining clinically available HDACi, such as FK228 or MS‐275, with ABT‐199 holds significant promise for improving clinical outcomes. Given DNTTIP1's involvement in DNA repair pathways, we investigated the therapeutic potential of PARPi—a class of agents with established efficacy in leukaemia.^59^, ^60^ This approach was further supported by preclinical evidence demonstrating synergistic interactions between PARPi and both HDACi and BCL2 antagonists.^61^, ^62^ Notably, our preclinical triple‐combination studies (PARPi‒HDACi‒BCL2i) demonstrate enhanced anti‐leukaemic activity versus dual regimens, warranting clinical evaluation to potentially establish a new therapeutic paradigm in acute leukaemia.

Epigenetic therapies have been extensively explored in cancers, yet systemic targeting of HDAC1 and HDAC2—two ubiquitously expressed and essential enzymes, remains challenging in the clinical setting due to their critical roles in normal cellular homeostasis. In contrast, our identification of the DNTTIP1‒HDAC1/2‒BMF axis, together with the preclinical success of the combination regimens derived from this finding, suggests that targeting DNTTIP1 may represent a more selective therapeutic strategy for acute leukaemia. Subsequent studies will aim to develop selective DNTTIP1‐targeting agents and explore their potential in combination therapies to overcome disease relapse. Notably, several areas warrant further exploration to fully harness the therapeutic potential of targeting DNTTIP1. First, the full spectrum of DNTTIP1‐mediated leukemogenic mechanisms beyond its interaction with HDAC1/2 remains to be elucidated, which will be essential for guiding the rational development of DNTTIP1‐targeted agents. Second, although our study supports a broad dependency on DNTTIP1 and HDAC1/2 across acute leukaemia, further investigation is needed to identify leukaemia subtypes with heightened sensitivity and to define the underlying mechanism. This precision in defining subtype‐specific requirements will be critical for future translational applications.

## 5 CONCLUSION

In conclusion, our findings establish DNTTIP1 as a critical oncogenic regulator with broad relevance across acute leukaemia. Through comprehensive investigation of DNTTIP1's biological functions in acute leukaemia, we have delineated its mechanistic role in disease pathogenesis, identifying BMF as a pivotal downstream effector. Unlike other extensively studied BH3‐only proteins such as PUMA and NOXA, BMF has remained enigmatic. Our work uncovers its dual role as a downstream effector of the DNTTIP1, uniquely bridging apoptosis and autophagy in acute leukaemia. This study not only characterises the functional significance of the DNTTIP1‒HDAC1/2‒BMF axis but also demonstrates the synergistic efficacy of HDACi and BH3 mimetics in acute leukaemia. Lastly, we pioneer the concept of triple‐combination therapy by incorporating PARPi, presenting a novel therapeutic paradigm. These preclinical insights provide a robust foundation for advancing targeted treatment strategies in acute leukaemia.

## AUTHOR CONTRIBUTIONS

Huitao Fan, Yongsheng Li, Yao Chen and Ruolin Xiu conceived and designed the study. Ruolin Xiu, Yuzhu Ma, Xinyu Li, Yue Wu and Meiling Sun conducted experiments and analysed data. Yueying Gao performed bioinformatics analyses. Yanhong Zhao, Shengjin Fan, Shuqian Xu and Qizhao Li contributed to patient samples. Ruolin Xiu drafted the manuscript, and Huitao Fan reviewed and revised it. Huitao Fan, Yongsheng Li, Shengjin Fan and Shuqian Xu supervised the work. All authors reviewed and approved the final manuscript.

## CONFLICT OF INTEREST STATEMENT

The authors declare they have no conflicts of interest.

## ETHICS STATEMENT

Human clinical specimens and associated data were obtained from the Department of Hematology at the First Affiliated Hospital of Harbin Medical University, with written informed consent from all participants and approved by the Research Ethics Committee and the Ethics Committee of the First Affiliated Hospital of Harbin Medical University (2022189). All animal experiments were approved by the Institutional Animal Care and Use Committee (IACUC 2024067) at the same institution and conducted in compliance with institutional and national guidelines for laboratory animal welfare.

## Supporting information



Supporting Information

## Data Availability

All sequencing data generated in this study have been deposited in the Gene Expression Omnibus under the following accession numbers: GSE305428, GSE305430, GSE305431, GSE305435 and GSE305437.
