# Supplementary material for: DNTTIP1 drives leukaemogenesis through MiDAC‐mediated epigenetic silencing of BMF
Source: Clin Transl Med. 2026 Jan 28;16(2):e70603. doi: 10.1002/ctm2.70603 (PMC12848531; doi:10.1002/ctm2.70603)
Supplement: Supplementary file 1 — Supporting Information [file CTM2-16-e70603-s001.pdf]

Supplementary Figures

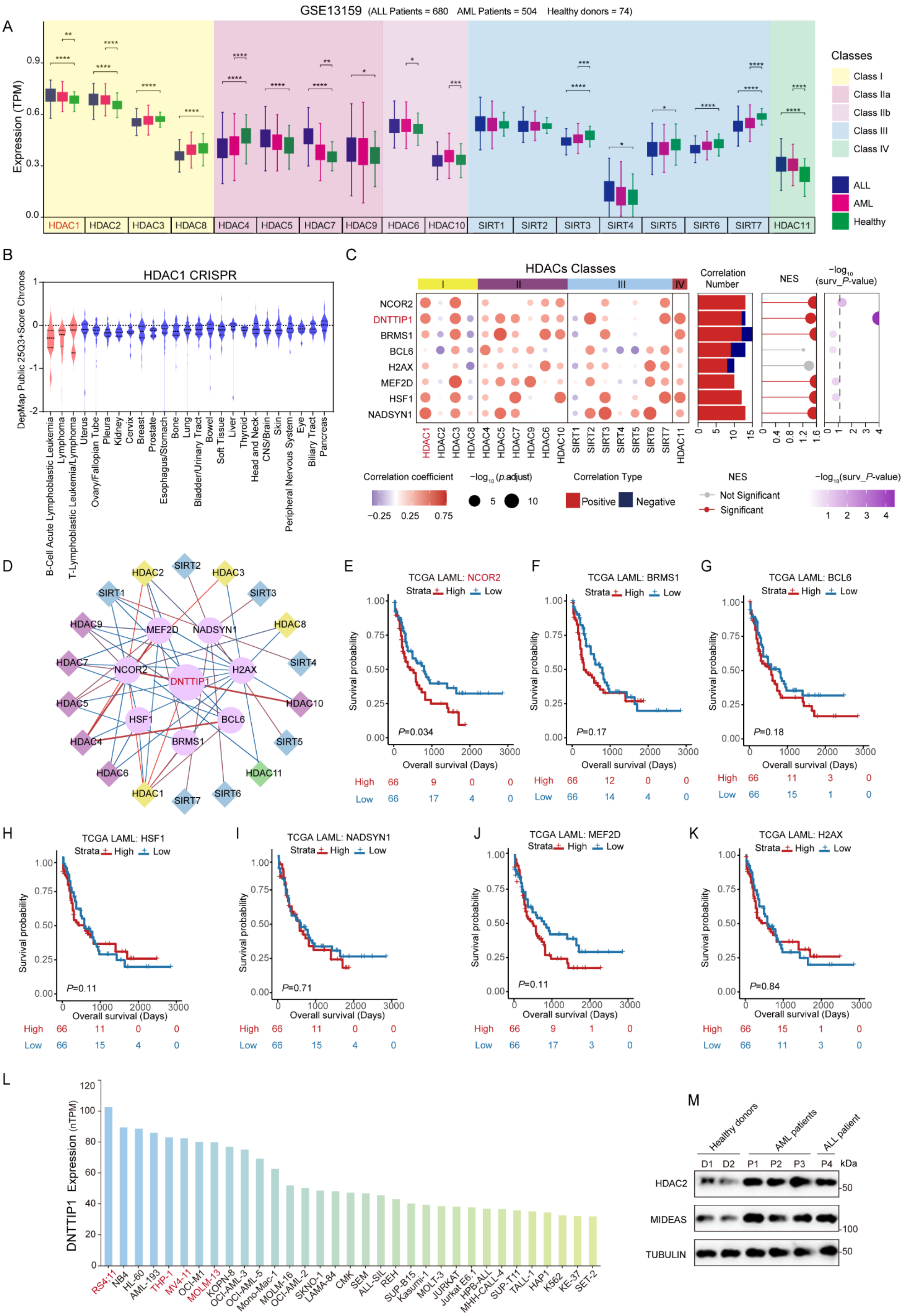

Figure S1. DNTTIP1 is a key interactor of HDAC1 and serves as a prognostic marker in acute leukemia.

A: Expression levels of HDACs classes from GEO dataset (GSE13159). Transcripts per million (TPM) was used to quantify gene expression.

B: Score of HDAC1 in the dependency map (DepMap) portal.

C: Dotplot visualization of the co-expression network between eight candidate genes and the HDAC family members.

Correlation Number: The number of HDAC genes that are significantly co-expressed with each candidate gene (FDR<0.05).

NES: Normalized enrichment score for HDAC pathway association (*P*<0.05).

Surv_P: Prognostic relevance of gene expression, where the dot size and position reflect the *P*-value, and dotted lines indicate the significance cutoff of *P*<0.05.

D: STRING-based protein-protein interaction network linking the eight genes identified in Figure 1B and HDAC family members.

E-K: Kaplan-Meier survival analysis of AML patients from the TCGA dataset, stratified by high versus low expression of seven genes (excluding DNTTIP1) using the median expression as the cutoff. Survival differences were evaluated using the log-rank test, with a significance threshold of *P*<0.05.

L: Quantitative analysis of DNTTIP1 transcript levels across acute leukemia cell lines using the Human Protein Atlas (HPA) database (https://www.proteinatlas.org/).

M: Western blot analysis of additional MiDAC complex components (HDAC2, MIDEAS) in bone marrow mononuclear cells (BMNCs) from acute leukemia patients (n=4) and healthy donors (n=2). A representative blot is shown.

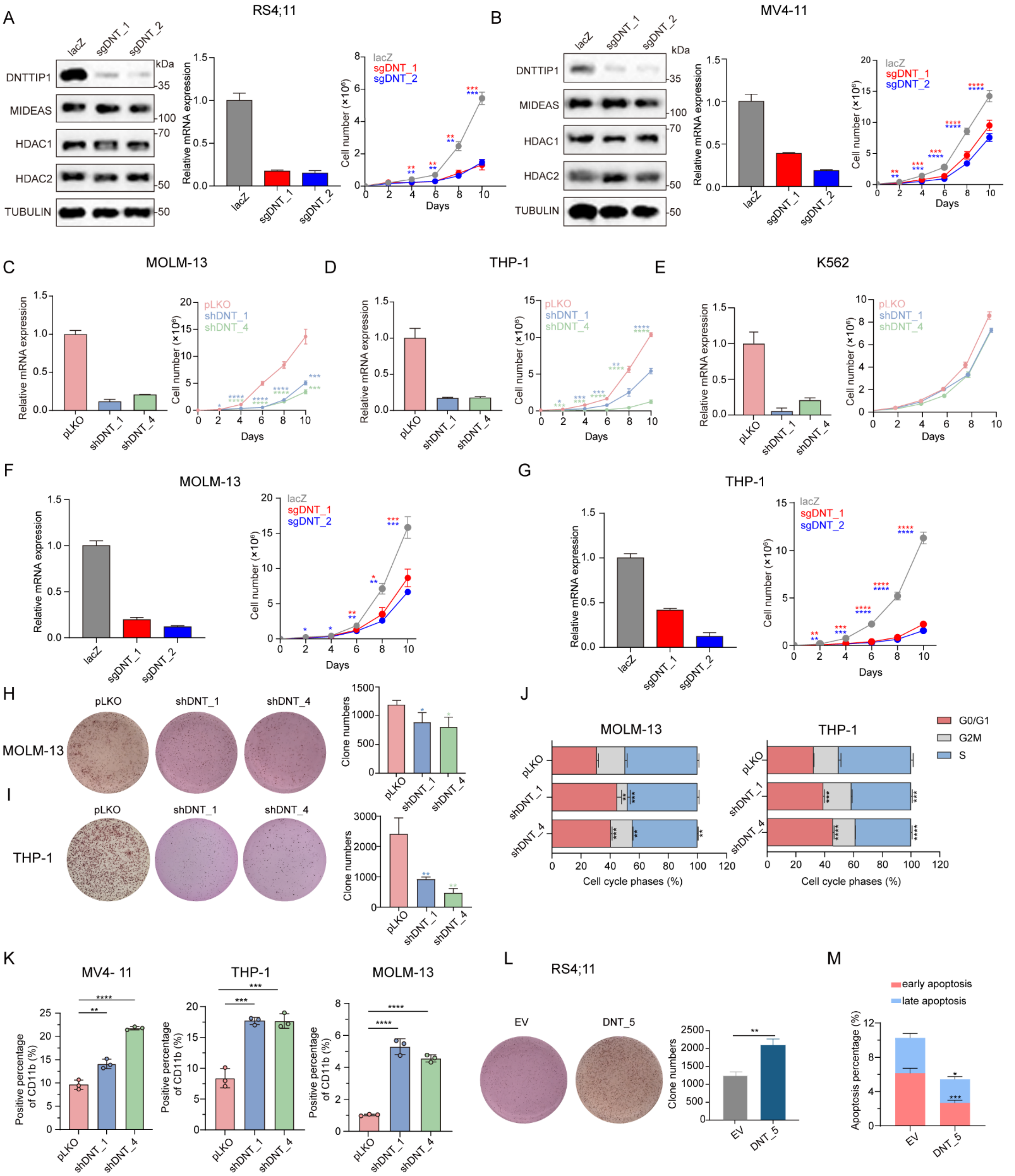

A
RS4;11
B
MV4-11
DNTTIP1
MIDEAS
HDAC1
HDAC2
TUBULIN
lacZ
sgDNT_1
sgDNT_2
Relative mRNA expression
Cell number (×10⁶)
Days
C
MOLM-13
D
THP-1
E
K562
pLKO
shDNT_1
shDNT_4
F
MOLM-13
G
THP-1
H
I
Clone numbers
J
G0/G1
G2M
S
Cell cycle phases (%)
K
MV4- 11
Positive percentage of CD11b (%)
L
RS4;11
EV
DNT_5
M
early apoptosis
late apoptosis
Apoptosis percentage (%)

Figure S2. DNTTIP1 is critical for acute leukemia cell survival and proliferation.

A, B: Immunoblotting of DNTTIP1 knockout (sgDNT_1 and sgDNT_2) efficiency and protein levels of MIDEAS, HDAC1, and HDAC2 after DNTTIP1 depletion (Left). RT-qPCR analyses of DNTTIP1 mRNA levels (Middle). Cell counting of RS4;11 (A) and MV4-11 (B) cells expressing sgDNT_1 and sgDNT_2 compared with control (lacZ) (Right) (n=3).

C-E: RT-qPCR (Left) analyses of DNTTIP1 knockdown efficiency in MOLM-13 (C), THP-1 (D), and K562 (E) cells using independent shRNAs (shDNT_1, shDNT_4). Cell counting of the indicated cell lines compared with control (pLKO) (Right) (n=3).

F, G: RT-qPCR (Left) analyses of DNTTIP1 knockout efficiency in MOLM-13 (F), THP-1 (G) cells using independent sgRNAs (sgDNT_1, sgDNT_2). Cell counting of the indicated cell lines compared with control (lacZ) (Right) (n=3).

H, I: Colony-forming unit (CFU) assay of MOLM-13 and THP-1 cells with DNTTIP1 knockdown (shDNT_1, shDNT_4) compared with control (pLKO) (n=3) (Left). Quantitative analysis of CFU assays (Right).

J: Flow cytometric analysis showing the cell cycle distribution of MOLM-13 and THP-1 cells with DNTTIP1 knockdown (shDNT_1, shDNT_4) compared with control (pLKO) (n=3).

K: Flow cytometric analysis of the myeloid differentiation marker CD11b in MV4-11, and THP-1, MOLM-13 cells following DNTTIP1 knockdown (shDNT_1, shDNT_4) compared with control (pLKO) (n=3).

L: Colony-forming unit (CFU) assay of RS4;11 cells with DNTTIP1 overexpression (DNTTIP1_5) compared with control (EV) (n=3) (Left). Quantitative analysis of CFU assays (Right).

M: Flow cytometric analysis of Annexin V/PI staining in RS4;11 cells with DNTTIP1 overexpression (DNTTIP1_5) compared with control (EV) (n=3).

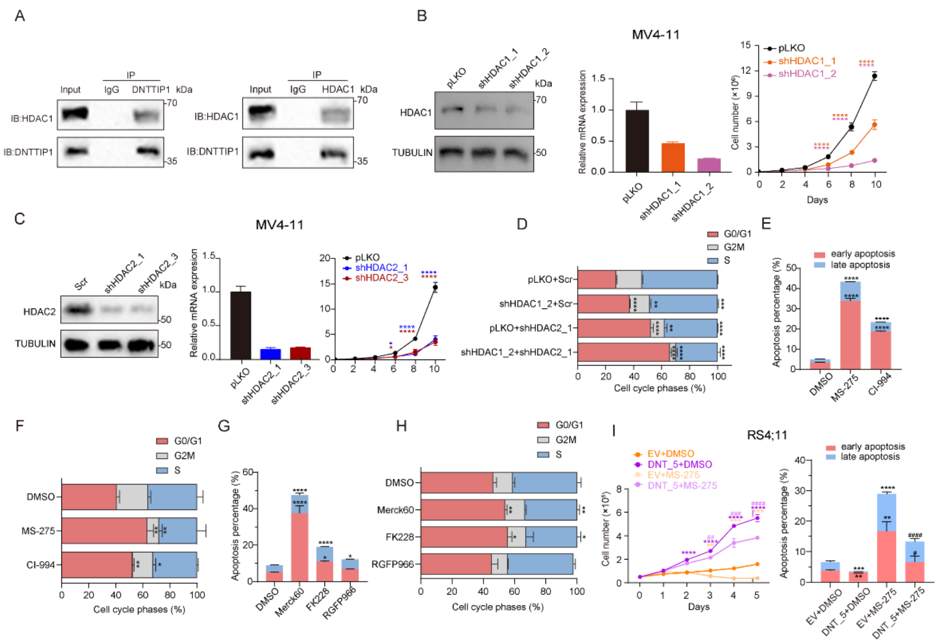

Figure S3. HDAC1/2 are critical for DNTTIP1-mediated leukemogenesis.

A: Reciprocal Co-IP analysis in RS4;11 cells demonstrated endogenous interaction between DNTTIP1 and HDAC1. (n=3).

B: Immunoblot (Left) and RT-qPCR (Middle) analyses demonstrating HDAC1 knockdown using two independent shRNAs (shHDAC1_1, shHDAC1_2). Cell counting of MV4-11 cells expressing shHDAC1_1, shHDAC1_2 compared with control (pLKO) (Right). (n=3).

C: Immunoblot (Left) and RT-qPCR (Middle) analyses demonstrating HDAC2 knockdown using two independent shRNAs (shHDAC2_1, shHDAC2_3). Cell counting of MV4-11 cells expressing shHDAC2_1, shHDAC2_3 compared with control (Scr) (Right). (n=3).

D: Flow cytometric analysis was performed to assess cell cycle distribution in RS4;11 cells with HDAC1/2 double knockdown versus control (pLKO+Scr). (n=3).

E, G: Flow cytometric analysis of Annexin V/PI staining in RS4;11 cells treated with indicated HDAC inhibitors (MS-275: 1 μM, CI-994: 2.5 μM) (E) or (Merck60: 2 μM, FK228: 1 nM, RGFP966: 10 μM) (G) for 48 h versus control (DMSO). (n=3).

F, H: Flow cytometric analysis was performed to assess cell cycle distribution in RS4;11 cells treated with indicated HDAC inhibitors (MS-275: 1 μM, CI-994: 2.5 μM) (F) or (Merck60: 2 μM, FK228: 1 nM, RGFP966: 10 μM) (H) for 48 h versus control (DMSO). (n=3).

I: Cell counting analysis showing the effect of MS-275 (0.5 μM, 48 h) treatment on cell proliferation (Left) and quantification of early (Annexin V^+ PI^-) and late (Annexin V^+ PI^+) apoptotic populations in DNTTIP1-overexpressing RS4;11 cells (Right). (n=3). DNT_5+MS-275 vs. DNT_5+DMSO denoted by #. All groups vs. EV+DMSO denoted by *. $^{\#}P$<0.05, $^{\#\#}P$<0.01, $^{\#\#\#}P$<0.001, $^{\#\#\#\#}P$<0.0001. $^{*}P$<0.05, $^{**}P$<0.01, $^{***}P$<0.001, $^{****}P$<0.0001.

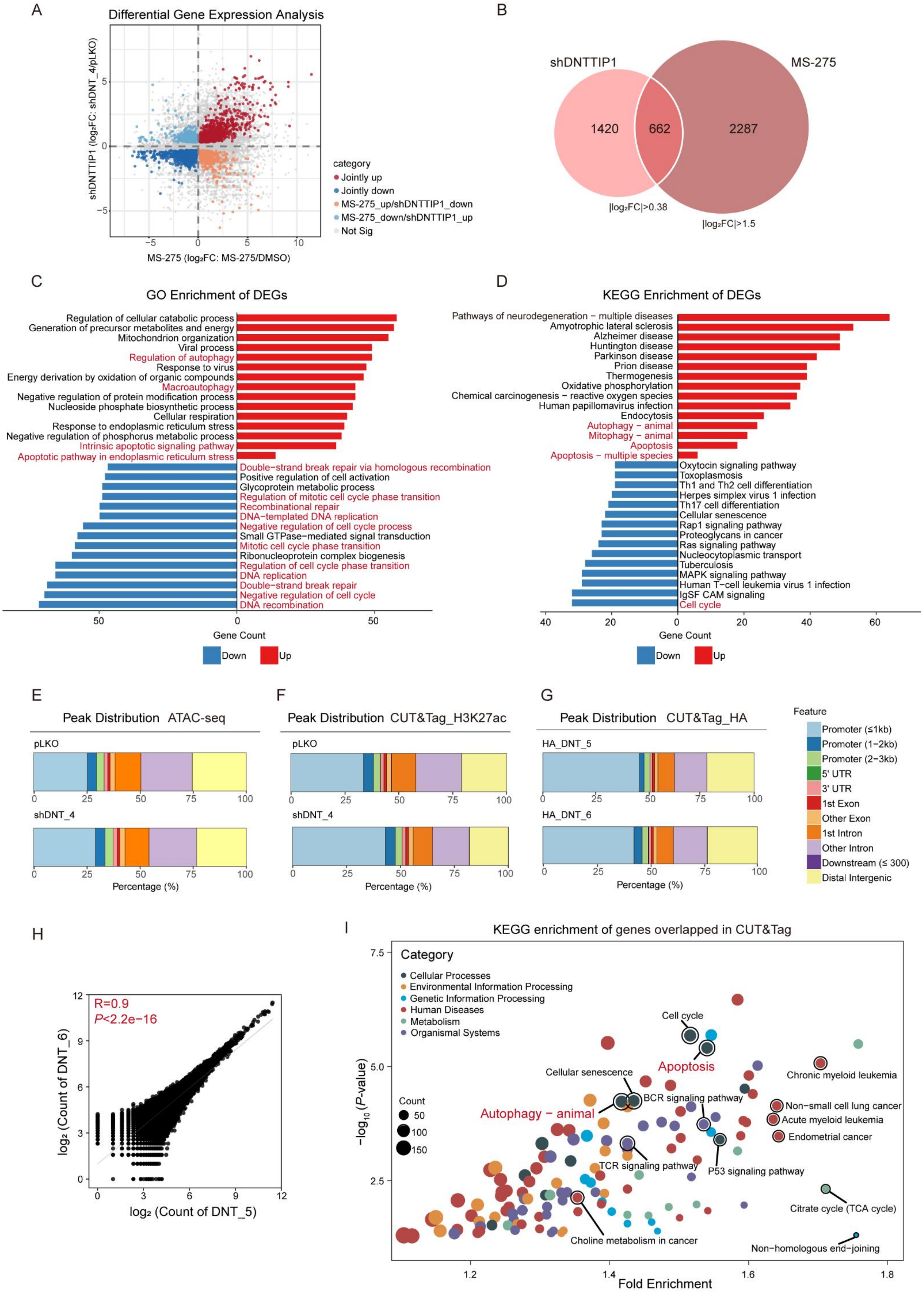
A
Differential Gene Expression Analysis
shDNTTIP1 (log₂FC: shDNT_4/pLKO)
MS-275 (log₂FC: MS-275/DMSO)
category
Jointly up
Jointly down
MS-275_up/shDNTTIP1_down
MS-275_down/shDNTTIP1_up
Not Sig
B
shDNTTIP1
MS-275
1420
662
2287
|log₂FC|>0.38
|log₂FC|>1.5
C
GO Enrichment of DEGs
Regulation of cellular catabolic process
Generation of precursor metabolites and energy
Mitochondrion organization
Viral process
Regulation of autophagy
Response to virus
Energy derivation by oxidation of organic compounds
Macroautophagy
Negative regulation of protein modification process
Nucleoside phosphate biosynthetic process
Cellular respiration
Response to endoplasmic reticulum stress
Negative regulation of phosphorus metabolic process
Intrinsic apoptotic signaling pathway
Apoptotic pathway in endoplasmic reticulum stress
Double−strand break repair via homologous recombination
Positive regulation of cell activation
Glycoprotein metabolic process
Regulation of mitotic cell cycle phase transition
Recombinational repair
DNA−templated DNA replication
Negative regulation of cell cycle process
Small GTPase−mediated signal transduction
Mitotic cell cycle phase transition
Ribonucleoprotein complex biogenesis
Regulation of cell cycle phase transition
DNA replication
Double−strand break repair
Negative regulation of cell cycle
DNA recombination
Gene Count
Down
Up
D
KEGG Enrichment of DEGs
Pathways of neurodegeneration − multiple diseases
Amyotrophic lateral sclerosis
Alzheimer disease
Huntington disease
Parkinson disease
Prion disease
Thermogenesis
Oxidative phosphorylation
Chemical carcinogenesis − reactive oxygen species
Human papillomavirus infection
Endocytosis
Autophagy − animal
Mitophagy − animal
Apoptosis
Apoptosis − multiple species
Oxytocin signaling pathway
Toxoplasmosis
Th1 and Th2 cell differentiation
Herpes simplex virus 1 infection
Th17 cell differentiation
Cellular senescence
Rap1 signaling pathway
Proteoglycans in cancer
Ras signaling pathway
Nucleocytoplasmic transport
Tuberculosis
MAPK signaling pathway
Human T−cell leukemia virus 1 infection
IgSF CAM signaling
Cell cycle
Gene Count
Down
Up
E
Peak Distribution ATAC-seq
pLKO
shDNT_4
Percentage (%)
F
Peak Distribution CUT&Tag_H3K27ac
pLKO
shDNT_4
Percentage (%)
G
Peak Distribution CUT&Tag_HA
HA_DNT_5
HA_DNT_6
Percentage (%)
Feature
Promoter (≤1kb)
Promoter (1−2kb)
Promoter (2−3kb)
5' UTR
3' UTR
1st Exon
Other Exon
1st Intron
Other Intron
Downstream (≤ 300)
Distal Intergenic
H
R=0.9
P<2.2e−16
log₂ (Count of DNT_6)
log₂ (Count of DNT_5)
I
KEGG enrichment of genes overlapped in CUT&Tag
Category
Cellular Processes
Environmental Information Processing
Genetic Information Processing
Human Diseases
Metabolism
Organismal Systems
Count
50
100
150
−log₁₀(P-value)
Fold Enrichment
Cell cycle
Apoptosis
Cellular senescence
Autophagy − animal
BCR signaling pathway
TCR signaling pathway
P53 signaling pathway
Chronic myeloid leukemia
Non-small cell lung cancer
Acute myeloid leukemia
Endometrial cancer
Citrate cycle (TCA cycle)
Non−homologous end−joining
Choline metabolism in cancer

Figure S4. DNTTIP1-HDAC1/2-mediated chromatin regulation modulates autophagy and apoptosis.

A: Volcano plot depicting DEGs under condition-specific gene expression analysis.

B: Venn diagram showing the overlap of DEGs in RS4;11 cells following DNTTIP1 knockdown (|logFC|>0.38, adjusted *P*<0.05) (Left) or MS-275 treatment (|logFC|>1.5, adjusted *P*<0.01) (Right), compared with control cells.

C, D: Bar graphs showing significantly enriched GO (C) and KEGG (D) pathways in DNTTIP1-depleted RS4;11 cells compared to controls. Red and blue bars represent pathways associated with upregulated and downregulated genes, respectively. Bar length indicates the number of genes (gene count).

E-G: Stacked bar chart illustrating the percentage distribution of regulatory peaks across the genome: ATAC-seq upon DNTTIP1 knockdown (E), H3K27ac CUT&Tag upon DNTTIP1 knockdown (F), and two independent single-clone HA-tagged DNTTIP1 CUT&Tag (G).

H: Scatter plot showing the high correlation of HA-DNTTIP1 CUT&Tag peaks between two independent single-clone.

I: KEGG pathway analysis of genes specifically enriched in the CUT&Tag peaks revealed significant enrichment in cancer-related signaling pathways, including apoptosis and autophagy.

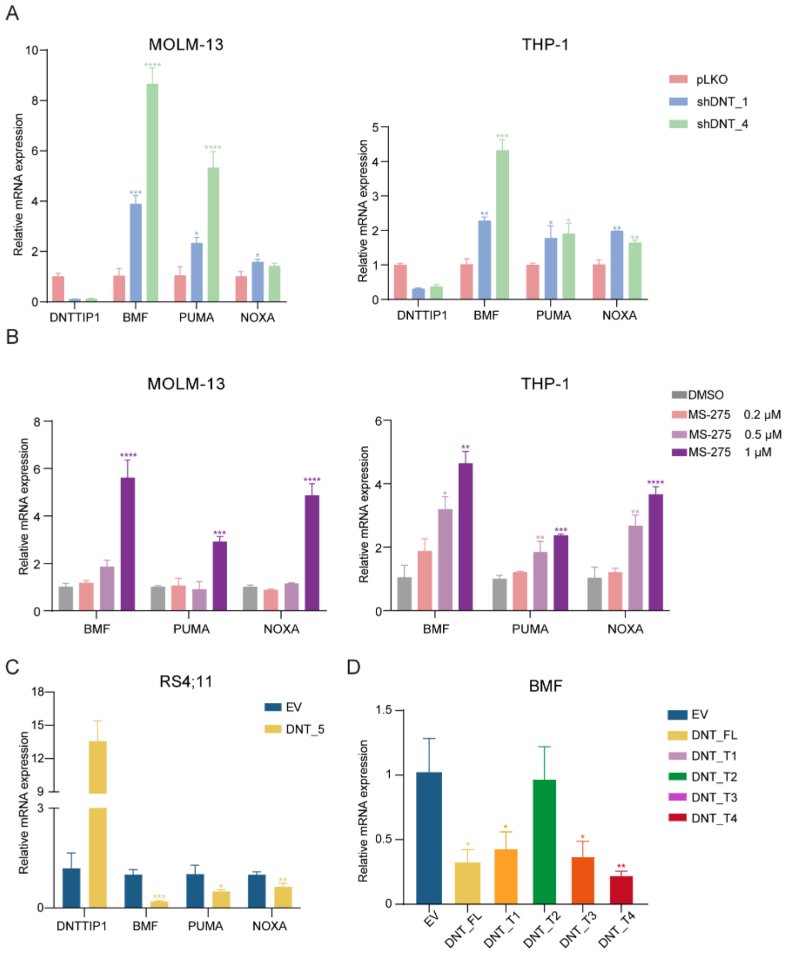

Figure S5. The DNTTIP1-HDAC1-BMF axis drives leukemogenesis.

A, B: RT-qPCR analysis of BH3-only genes (BMF, PUMA, and NOXA) mRNA expression following DNTTIP1 knockdown (A) or MS-275 treatment (B) in leukemia cells.

C: RT-qPCR analysis of BH3-only genes (BMF, PUMA, and NOXA) mRNA expression following DNTTIP1 overexpression in RS4;11 cells.

D: RT-qPCR analysis of BH3-only genes (BMF, PUMA, and NOXA) mRNA expression in RS4;11 cells expressing DNTTIP1 full-length or truncated constructs.

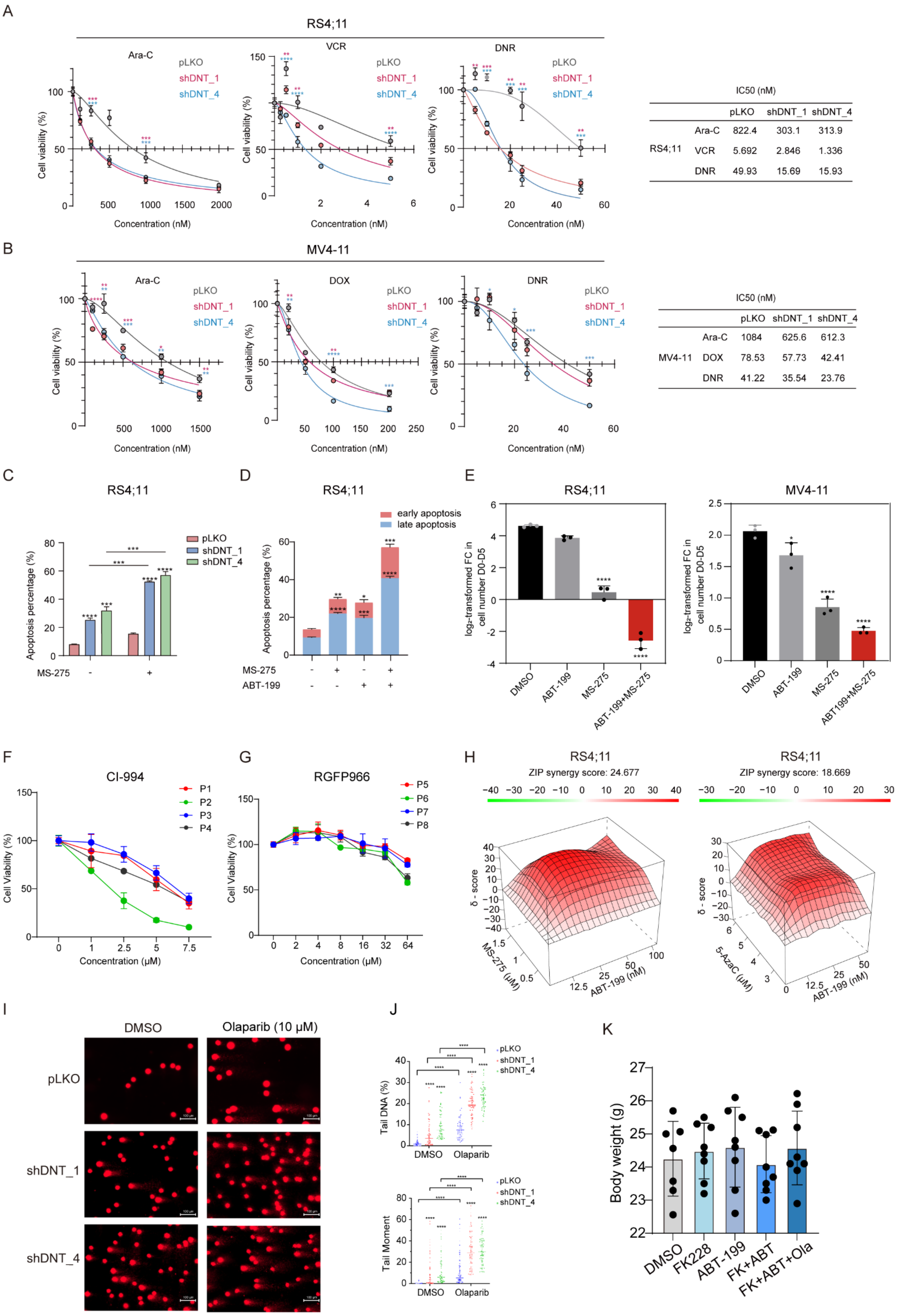

IC50 (nM)

		pLKO	shDNT_1	shDNT_4
RS4;11	Ara-C	822.4	303.1	313.9
	VCR	5.692	2.846	1.336
	DNR	49.93	15.69	15.93

IC50 (nM)

		pLKO	shDNT_1	shDNT_4
MV4-11	Ara-C	1084	625.6	612.3
	DOX	78.53	57.73	42.41
	DNR	41.22	35.54	23.76

Figure S6. DNTTIP1 loss enhances chemosensitivity, and HDACi/BCL2i/PARPi triple therapy shows potent synergy in acute leukemia.

A, B: Anti-leukemic activity of Ara-C, DNR, VCR, and DOX in control (pLKO) and DNTTIP1 knockdown (shDNT_1, shDNT_4) RS4;11 (A) and MV4-11 (B) cells. Cells were treated with a gradient of chemotherapy drug concentrations, and cell viability was determined after 48 hours using the CellTiter-Glo (CTG) assay (Left). The table displays the IC50 values of chemotherapy drugs for RS4;11 and MV4-11 cell proliferation. The DNTTIP1 knockdown had lower IC50 values compared with the control (Right).

C: Flow cytometric analysis of Annexin V/PI staining in RS4;11 cells with DNTTIP1 knockdown (shDNT_1, shDNT_4) versus control (pLKO) upon MS-275 (0.5 μM, 48 h)/DMSO treatment. (n=3).

D: Flow cytometric analysis of Annexin V/PI staining in RS4;11 cells treated with ABT-199 and/or MS-275 (MS-275: 1 μM, ABT-199: 100 nM, 48 h) versus control (DMSO). (n=3).

E: RS4-11 and MV4-11 cells were treated with ABT-199 and/or MS275 for 5 days, and the relative change in cell number was assessed (D5 vs. D0). The concentration of MS275 was 0.5 μM, while ABT-199 was used at 50 nM. Quantitative data are presented as the mean±s.d. from at least three independent experiments and analyzed by Student's t-test. **P*<0.05, ***P*<0.01, ****P*<0.001, *****P*<0.0001.

F, G: The viability of primary acute leukemia BMNCs was determined using the CTG assay following treatment with CI-994 (F) or RGFP966 (G) at multiple concentrations for 48 h.

H: RS4;11 cells were exposed to ABT-199 in combination with MS-275 (Left) or 5-AzaC (Right) at multiple concentrations for 48 hours.

I, J: Representative alkaline comet assay images (I) and quantitative analysis (J) of control and DNTTIP1 knockdown cells treated with Olaparib (10 μM, 48 h). Scale bar, 100 μm.

K: Body weight of PDX mice in the five groups at the end of the 3-week treatment period. Data are presented as mean±SD (n=8 per group).

Supplementary Tables

Table S1. Clinical features of acute leukemia cases for bone marrow samples

No.	Sex	Age (years)	Immunotype	Cytogenetic abnormality	Fusion gene	Mutant	Disease condition	Percentage of blast cells (%)
D1	F	27	(-)	(-)	(-)	(-)	Healthy	(-)
D2	M	49	(-)	(-)	(-)	(-)	Healthy	(-)
P1	F	51	Myeloid	(-)	(-)	(-)	Newly diagnosis	72
P2	F	50	Myeloid	(-)	(-)	DNMT3A, KIT, PTPN11, NRAS, NPM1, IDH2	Newly diagnosis	69.5
P3	F	76	Myeloid	(-)	CBFβ-MYH11	(-)	Newly diagnosis	27
P4	M	35	Common B cell	(-)	(-)	(-)	Newly diagnosis	94.5
P5	M	57	Myeloid	(-)	(-)	FLT3-ITD, DNMT3A, NPM1, SETBP1	Newly diagnosis	32.5
P6	M	72	Myeloid	(-)	(-)	RUNX1, IDH2, DNMT3A, ASXL1	Newly diagnosis	89.5
P7	F	25	Myeloid	47, XX,+8	(-)	FLT3-TKD,BCORL1, KMTA2	Newly diagnosis	39
P8	F	58	Pre-T cell	(-)	(-)	FLT3-TKD, NRAS	Newly diagnosis	88
PDX	M	70	Common B cell	46, XY, -7, der (9) t (7; 9) (q11; p12) t(9; 22) (q34.1; q11.2), der (22) t (9; 22) + der (22) t (9; 22) [20]	BCR-ABL1 Ph+	(-)	Newly diagnosis	67.5

Table S2. Antibodies for Western blotting, immunofluorescence, ChIP and CUT&Tag

Antibodies	Source	Application
DNTTIP1/TdTIF1	Santa Cruz Biotechnology, sc-166296	WB, PLA
MIDEAS/ELMSAN1	Santa Cruz Biotechnology, sc-514710	WB
HDAC1	Santa Cruz Biotechnology, sc-81598	WB, Co-IP
HDAC1	Proteintech, 10197-1-AP	ChIP-qPCR, PLA
HDAC2	Abcam, ab7029	WB, Co-IP
H3K27ac	Abcam, ab4729	WB, ChIP-qPCR, CUT&Tag
Histone H3	Abcam, ab1791	WB
BMF	Abways, CY8053	WB
SP1	Abcam, ab231778	WB, Co-IP, ChIP-qPCR
SQSTM1/P62	Santa Cruz Biotechnology, sc-48402	WB
LC3B	Cell Signaling Technology, 3868	WB
PARP	Proteintech, 22999-1-AP	WB
Caspase 3	Santa Cruz Biotechnology, sc-56053	WB
Caspase 9	Santa Cruz Biotechnology, sc-56076	WB
TdT	Abways, CY8847	WB
Beta Tubulin	Proteintech, 80713-1-RR	WB
HA	Cell Signaling Technology, 3724	WB, Co-IP, ChIP-qPCR, CUT&Tag
GST	Proteintech, .10000-0-AP	WB
His	Proteintech, 66005-1-Ig	WB
Mouse IgG	Proteintech, B900620	Co-IP
Rabbit IgG control Polyclonal antibody	Proteintech, 30000-0-AP	Co-IP
HRP-conjugated Goat Anti-Mouse IgG(H+L)	Proteintech, SA00001-1	WB
HRP-conjugated Goat Anti-Rabbit IgG(H+L)	Proteintech, SA00001-2	WB
IgG Negative Control Antibody	EpiCypher, 13-0042	CUT&Tag

Table S3. Plasmids and primer sequences

Application	Gene	Forward primer (5'-3')	Reverse primer (5'-3')
RT	GAPDH	TGCACCACCAACTGCTTAGC	GGCATGGACTGTGGTCATGAG
RT	DNTTIP1	CCCAGCTCGCCTGAATGAAT	GCTGCATACTTAAAGAGGTGTGG
RT	BMF	GGAGCTGGAGGATGATGTGT	CAGTGGGTGAGAGGGAAGAG
RT	NOXA	CTGGAAGTCGAGTGTGCTAC	AGGTTCCTGAGCAGAAGAGT
RT	PUMA	GCGGGGAGGAGGAACAGT	TCCCATGATGAGATTGTACAGGA
RT	HDAC1	TGTCGGAGTACAGCAAGCAG	TGCCTCGGACTTCTTTGCAT
RT	HDAC2	GCCACTGCCGAAGAAATGAC	TCCAGCCCAATTAACAGCCA

Application	Gene	Forward start	Forward primer (5'-3')	Reverse start	Reverse primer (5'-3')
ChIP-qPCR	BMF	-192 bp	TCACTCGCCATTGGTCAGTC	-101 bp	TCCAAAATACGCCTGCTCGG

Application	Gene	Forward primer (5'-3')	Reverse primer (5'-3')
shRNA	DNTTIP1 (shDNT_1)	CCGGATGGATCTCAGCGGCATTAAGCTCGA<br>GCTTAATGCCGCTGAGATCCATTTTTT	AATTAAAAAATGGATCTCAGCGGCATTAAGC<br>TCGAGCTTAATGCCGCTGAGATCCAT
shRNA	DNTTIP1 (shDNT_4)	CCGGGCGAGTTTGTAGTGACTCACTCTCG<br>AGAGTGAGTCACTACAAACTCGCTTTTT	AATTAAAAAGCGAGTTTGTAGTGACTCACTC<br>TCGAGAGTGAGTCACTACAAACTCGC
shRNA	HDAC1 (shHDAC1_1)	CCGGCCGCAAGAACTCTTCCAACTTCTCGA<br>GAAGTTGGAAGAGTTCTTGCGGTTTTTG	AATTCAAAAACCGCAAGAACTCTTCCAACTT<br>CTCGAGAAGTTGGAAGAGTTCTTGCGG
shRNA	HDAC1 (shHDAC1_2)	CCGGGCTGCTCAACTATGGTCTCTACTCGA<br>GTAGAGACCATAGTTGAGCAGCTTTTTG	AATTCAAAAAGCTGCTCAACTATGGTCTCTACT<br>CGAGTAGAGACCATAGTTGAGCAGC
shRNA	HDAC2 (shHDAC2_1)	CCCTTGGAGAAAAGCCTTGTTTCAGACTGA<br>TATGGCTGTTAATTTCAAGAGAATTAA	CTCGTGAAGCGAGCTTATAAAAAACAGACT<br>GATATGGCTGTTAATTCTCTTGAAA
shRNA	HDAC2 (shHDAC2_3)	CCCTTGGAGAAAAGCCTTGTTTGTTGCTCG<br>ATGTTGGACATATTTCAAGAGAATATG	CTCGTGAAGCGAGCTTATAAAAAAGTTGCT<br>CGATGTTGGACATATTCTCTTGAAA
shRNA	BMF (shBMF_1)	CCCTTGGAGAAAAGCCTTGTTTGCAACATC<br>AAGCAGAGGTACATTCAAGAGATGTAC	CTCGTGAAGCGAGCTTATAAAAAAGCAACA<br>TCAAGCAGAGGTACATCTCTTGAAT
shRNA	BMF (shBMF_2)	CCCTTGGAGAAAAGCCTTGTTTCAACCTTG<br>CTTTGAATGGAGATTCAAGAGATCTCC	CTCGTGAAGCGAGCTTATAAAAAACAACCT<br>TGCTTTGAATGGAGATCTCTTGAAT
shRNA	sh*Dnttip1*	CCCTTGGAGAAAAGCCTTGTTTGATGACCA<br>CAAGTTTCACAGATTCAAGAGATCTGT	CTCGTGAAGCGAGCTTATAAAAAAGATGAC<br>CACAAGTTTCACAGATCTCTTGAAT
sgRNA	sgDNTTIP1 (sgDNT_1)	Target: CATAATGATAAAGCACCGGC	
sgRNA	sgDNTTIP1 (sgDNT_2)	Target: TTGTTGTCATCTGTGAGCGG	
sgRNA	sgSP1 (sgSP1_1)	Target: GCTACTTCGAGCCTGTGAAA	
sgRNA	sgSP1 (sgSP1_2)	Target: AGAATCGCACAGTCTCTGGT	
siRNA	DNTTIP1 (siDNT_1)	CGCUCACAGAUGACAACAAtt	UUGUUGUCAUCUGUGAGCGtt
siRNA	DNTTIP1 (siDNT_2)	AGACUGUCUUCAACAAGUAtt	UACUUGUUGAAGACAGUCUtt

Supplementary Materials and Methods

RNA Extraction and Quantitative Real-time PCR (RT-qPCR)

RNA was extracted using the RNA purification kit (TransGen Biotech, ER101-01). One microgram of purified RNA was subjected to reverse transcription using the cDNA Synthesis SuperMix (YEASON, 11141ES60). RT-qPCR was performed on the BIOER Real-Time PCR System using Universal SYBR Green Master Mix (YEASON, 11184ES08). The relative expression of target genes was calculated using the $2^{-\Delta\Delta Ct}$ method, with glyceraldehyde-3-phosphate dehydrogenase (GAPDH) serving as the reference gene. Primer sequences are available in the Supplementary Table 3.

Western blotting (WB)

The total cell lysates were prepared using RIPA lysis buffer (Beyotime, P0013B, Shanghai, China) supplemented with protease inhibitor cocktail (Proteintech, PR20016, Wuhan, China). Protein concentrations were measured using the Enhanced BCA Protein Assay Kit (Beyotime, P0009, Shanghai, China). Equal amounts of protein were resolved on 10% or 12.5% SDS-PAGE gel and transferred to polyvinylidene difluoride (PVDF) membrane (Millipore, 3010040001, USA). The membranes were blocked with 5% non-fat milk at ambient temperature for 1 hour and subsequently probed with primary antibodies through overnight incubation at 4°C. After washing with 1×PBST three times, the membranes were incubated with horseradish peroxidase (HRP)-conjugated secondary antibodies for 2 hours at room temperature. β-Tubulin and Histone 3 were used as the loading control, respectively. Ultimately, the protein bands were visualized with Super ECL Plus Detection Reagents (Epizyme, SQ201L, Shanghai, China). The protein expression levels were visualized using a Chemiluminescent Imaging System (Tanon, 5200, Shanghai, China).

Co-immunoprecipitation (Co-IP)

Cells was harvested and lysed in ice-cold modified Co-IP lysis buffer (Proteintech, PR20037, Wuhan, China) supplemented with DNase I (Beyotime, D7073, Shanghai, China) on ice for 30 minutes. The

lysates were purified by centrifugation at 14000×g for 10 min at 4°C, and then pre-cleared by incubation with BeyoMag Protein A/G magnetic beads (Beyotime, P2108, Shanghai, China) for 30 minutes with gentle rotation at 4°C to reduce non-specific binding. Control IgG or primary antibodies were added to the collected cell supernatant and incubated on a rocking platform overnight at 4°C. The samples were supplemented with Protein A/G magnetic beads and rotated continuously at 4°C for 2 hours to facilitate the capture of antibody-antigen complexes. Following capture of the complex using a magnet, the beads were washed for four times with Co-IP lysis buffer, and the bound proteins were analyzed by Western blotting.

Flow cytometry

For cell apoptosis assay, cells were stained with 5 μL Annexin V-FITC and 5 μL PI for 25 minutes at room temperature in the dark according to the instruction of Annexin V-FITC/PI Apoptosis Assay Kit (Elabscience, E-CK-A211, Wuhan, China). For cell differentiation assay, cells were stained with FITC-labeled anti-CD11b (Elabscience, E-AB-F1081C, Wuhan, China) for flow cytometry analysis. For cell cycle analysis, 5×10^5 cells were resuspended in PBS, and pre-cooled absolute ethanol was slowly added dropwise to reach a final concentration of 80%, followed by fixation at -20°C overnight. Cells were washed and resuspended in 100 μg/mL RNase A solution for 30 minutes at 37°C, followed by incubation with 40 μg/mL propidium iodide (PI) at 4°C for 30 minutes in the dark, according to the instruction of Cell Cycle Assay Kit (Elabscience, E-CK-A351, Wuhan, China). Flow cytometric analysis was performed on the BD Accuri C6 flow cytometer (BD Biosciences,USA), and the data were analyzed with FlowJo software (version 10.8.1) and ModFit LT (version 5).

Wright-Giemsa

Cells were collected and washed with pre-cooled PBS three times and resuspended in plasma. Cell suspension was smeared onto microscope slides (CITOTEST, Jiangsu, China) and stained with Wright-Giemsa staining fluid (Baso, BA4017, Zhuhai, China), followed by gentle washing and

observation under a light microscope and photographed.

Colony-forming unit (CFU) assay

Cells were seeded in 6-well plates with 1.5 ml of medium containing 10% serum and 0.36% agar in the upper layer, and 2 ml of the same medium containing 0.75% agar in the lower layer. Viable colonies were stained with iodonitrotetrazolium chloride (V900870, Sigma-Aldrich, Shanghai, China) for 24 hours. The stained colonies were imaged and quantified using ImageJ software.

Cell viability assay

Cells were reseeded into 96-well plates and treated with the indicated compounds. Cell viability was then assessed using the CellTiter-Glo Luminescent Cell Viability Assay (Promega, G7570, USA) according to the manufacturer's instruction. Bioluminescence signals were measured with a SpectraMax i3 Multimode Microplate Reader (Molecular Devices, USA).

RNA sequencing (RNA-seq) and data analysis

Total RNA was isolated as previously described. RNA integrity was evaluated using the RNA Nano 6000 Assay Kit of the Bioanalyzer 2100 system (Agilent Technologies, CA, USA), with all samples exhibiting RIN (RNA Integrity Number)>8.0. Indexed libraries were constructed and clustered on a cBot Cluster Generation System using TruSeq PE Cluster Kit v3-cBot-HS (Illumina, San Diego, USA), followed by 150 bp paired-end sequencing on the Illumina NovaSeq 6000 platform.[1]

Raw sequencing reads were quality-filtered using fastp (v.0.23.4) to remove adapter sequences and low-quality bases, followed by alignment to the GRCh38 human reference genome using STAR (v2.7.11) with default parameters. Gene-level read counts were quantified using featureCounts (v.2.0.6), and differential expression analysis was performed with the DESeq2 (v.1.40.2) package in R, where genes with $P<0.05$ were considered statistically significant. The function enrichment analyses, including Gene ontology (GO) enrichment analysis for the biological processes terms, Kyoto Encyclopedia of Genes and Genomes (KEGG) enrichment analysis and gene set enrichment

analysis (GSEA), were conducted via clusterProfiler package (4.14.5).

Cleavage under targets and tagmentation (CUT&Tag) and data analysis

CUT&Tag assay was performed using the Hyperactive Universal CUT&Tag Assay Kit for Illumina (Vazyme, TD904, China). Briefly, 1×10^5 fresh cells were bound to ConA magnetic beads (10 min, RT) and incubated overnight at 4°C with primary antibodies (anti-HA, anti-H3K27ac, and IgG as negative control). The next day, samples were sequentially incubated with secondary antibody (1 h, RT) and pA-Tn5 transposase complex (1 h, RT). After extensive washing, DNA was fragmented and eluted, followed by library preparation using the TruePrep Index Kit V2 for Illumina (Vazyme, TD202, China) and sequencing on the Illumina NovaSeq 6000 platform.

Raw FASTQ files underwent quality control with fastp (v.0.23.4), followed by alignment to the GRCh38 human genome using bowtie2 (v.2.5.4). Aligned reads (BAM format) were converted to bigWig coverage files using deeptools (v.3.5.5), and peaks were called with MACS3 (v.3.0.0) for differential analysis. Visualization of sequencing tracks utilized the Integrative Genome Viewer (IGV) (v.2.16.0), while chromVAR (v.1.30.1) quantified CUT&Tag signals via the getCounts function. The heatmaps and averaged plotting of CUT&Tag signals were generated by computeMatrix and plotHeatmap functions from deeptools (v.3.5.5). The significant peaks were annotated using ChIPseeker (v.1.42.1), and function enrichment analyses were performed by clusterProfiler package (v.4.14.5).

Assay for transposase-accessible chromatin using sequencing (ATAC-seq) and data analysis

ATAC-seq assay was performed using the Hyperactive ATAC-seq Library Prep Kit for Illumina (Vazyme, TD711, China). Briefly, 1×10^5 fresh cells were washed with ice-cold TW Buffer and lysed to release nuclei. After centrifugation (500×g, 5 min, 4°C), nuclei were resuspended in transposition mix (37°C, 30 min) for simultaneous fragmentation and tagmentation. Transposed DNA was purified using magnetic DNA Extract Beads, PCR-amplified with indexed primers, and processed into

sequencing libraries using the TruePrep Index Kit V2 for Illumina (Vazyme, TD202, China). Libraries were sequenced on the Illumina NovaSeq 6000 platform.

In this analysis, raw sequencing data underwent quality control using fastp (v.0.23.4), followed by alignment to the human reference genome (hg38) with Bowtie2 (v.2.5.4). SAM-formatted alignment files were converted to BAM format using samtools (v.1.21), and PCR duplicates were removed via Picard MarkDuplicates function (v.3.4.0). Read coverage profiles (BigWig format) were generated using deeptools (v.3.5.5). Peak calling and differential peak analysis were performed with MACS3 (v.3.0.0), and results were visualized in IGV (v.2.16.0). The computeMatrix and plotHeatmap functions from deeptools (v.3.5.5) were applied to generate heatmaps and averaged plotting of ATAC signals. Significant peaks were annotated with ChIPseeker package (v.1.42.1), and functional enrichment analysis was conducted using clusterProfiler package (v.4.14.5). Enrichment of motifs was calculated by 'findMotifsGenome.pl' function of HOMER (Hyper geometric Optimization of Motif Enrichment), and visualized via ggplot2 package (v.3.5.2).

References

1. Chepelev I, Wei G, Tang Q, Zhao K. Detection of single nucleotide variations in expressed exons of the human genome using RNA-Seq. *Nucleic Acids Res*. Sep 2009;37(16):e106. doi:10.1093/nar/gkp507
